# Cytotoxic Properties of C_17_ Polyacetylenes from the Fresh Roots of *Panax ginseng* on Human Epithelial Ovarian Cancer Cells

**DOI:** 10.3390/molecules27207027

**Published:** 2022-10-18

**Authors:** Ranhee Kim, So-Ri Son, Na-Kyung Lee, Ji-Young Kim, Gami An, Jung-Hye Choi, Dae Sik Jang

**Affiliations:** 1 Department of Life and Nanopharmaceutical Sciences, Graduate School, Kyung Hee University, Seoul 02447, Korea; 2 Department of Biomedical and Pharmaceutical Sciences, Graduate School, Kyung Hee University, Seoul 02447, Korea; 3 Department of Oriental Pharmaceutical Science, College of Pharmacy, Kyung Hee University, Seoul 02447, Korea

**Keywords:** cytotoxicity, human ovarian cancer, *Panax ginseng*, polyacetylenes

## Abstract

Although C_17_ polyacetylenes from *Panax ginseng* exhibit cytotoxic properties against various tumor cells, there have been few experiments on epithelial ovarian carcinoma cells. This study aimed to investigate the cytotoxic effects of C_17_ polyacetylenes from *P. ginseng* against ovarian cancer cell lines. Four unreported (**1**–**4**) and fifteen known (**5**–**19**) C_17_ polyacetylenes were obtained from the roots of *P*. *ginseng* using repeated chromatography (open column, MPLC, and preparative HPLC). The chemical structures of all the compounds were determined by analyzing their spectroscopic data (NMR, IR, and optical rotation) and HR-MS. The structures of new polyacetylenes were elucidated as (3*S*,8*S*,9*R*,10*R*)-(-)-heptadeca-9,10-epoxy-4,6-diyne-3,8-diyl diacetate (**1**), (3*S*,8*S*,9*R*,10*R*)-(−)-heptadeca-1-en-9,10-epoxy-4,6-diyne-3,8-diyl diacetate (**2**), (−)-haptadeca-9,10-epoxy-8-methoxy-4,6-diyne-3,11-diol (**3**), and (3*R*,9*R*,10*R*)-(+)-3-acetoxy-9,10-dihydroxyheptadeca-1-en-4,6-diyne (**4**), named ginsenoynes O, P, and Q, and 3-acetyl panaxytriol, respectively. Subsequently, in vitro experiments on A2780 and SKOV3 human epithelial ovarian carcinoma cells were performed to assess the cytotoxic properties of the isolates. Among the isolates, panaquinquecol 4 (**15**) exhibited the most remarkable cytotoxic effects on both human ovarian cancer cells A2780 (IC_50_ value of 7.60 μM) and SKOV3 (IC_50_ value of 27.53 μM). Therefore, C_17_ polyacetylenes derived from *P*. *ginseng* may warrant further investigation for their therapeutic potential in epithelial ovarian cancer.

## 1. Introduction

Ovarian cancer, identified as a group of malignant neoplasms, poses a lethal threat to women worldwide [1]. It is the third most common gynecological malignancy, with around 31 million officially recorded cases and 20 million deaths in 2020 [2]. Even the most frequent type of ovarian cancer, epithelial cancer, has a poor prognosis due to the asymptomatic characteristics of its early stages, with a 5-year relative survival rate of only 30% [3,4]. Currently, the common treatment procedure is to first have cytoreductive surgery and to then proceed with platinum-based chemotherapy [5]. However, platinum-based drugs damage normal cells substantially, which can result in organ dysfunction syndrome [6]. Platinum resistance in the treatment of ovarian cancer is also the main factor decreasing survival rate and increasing the risk of relapse [7]. Therefore, novel drugs are urgently required for the effective treatment of ovarian cancer.

Numerous anti-cancer agents derived from traditional medicines have been developed as alternatives to platinum-based chemotherapy with reduced toxic side effects and resistance [8]. *Panax ginseng* Meyer (Araliaceae) and its constituents have anti-tumor effects in various cancer cell lines, including ovarian cancer [9]. The unique saponin, 20-(*S*)-ginsenoside Rg3, exhibited anti-tumor effects in ovarian cancer cell lines (SKOV3 and A2780) and in vivo models by inhibiting the Warburg effect caused by reduced lactate synthesis or glucose metabolism [10]. In addition to ginsenosides, a recent review has indicated that C_17_ polyacetylenes, such as panaxydiol and panaxytriol in *Panax* species, effectively suppress cell proliferation in several cancer cell lines, inducing cell cycle arrest at multi-phases [11]. Although C_17_ polyacetylenes have potential cytotoxic properties and are abundant among the polyacetylenes found in *P*. *ginseng*, few investigations on their activity in human ovarian cancer have been conducted. In our preliminary study, we identified the cytotoxic property of polyacetylene-enriched extracts from the fresh roots of *P*. *ginseng* on epithelial ovarian cancer cells. Therefore, the present research aimed to identify C_17_ polyacetylenes in *P*. *ginseng* with cytotoxic effects on human ovarian cancer cell lines.

**Figure 1:**
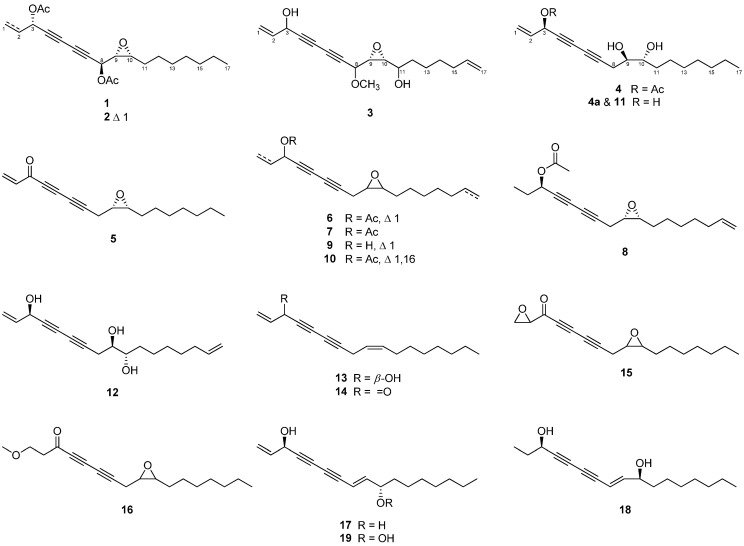
Chemical structures of compounds **1**–**19** isolated from the roots of *P*. *ginseng*.

The present study used repeated chromatography to isolate four newly reported C_17_ polyacetylenes (**1**–**4**) and 15 known compounds (**5**–**19**) from a 70% acetone extract of *P*. *ginseng* roots (Figure 1). The cytotoxicity of the isolated compounds was evaluated in human ovarian cancer cell lines A2780 and SKOV3.

## 2. Results

### 2.1. Structure Elucidation of Isolated Compounds

**Figure 2:**
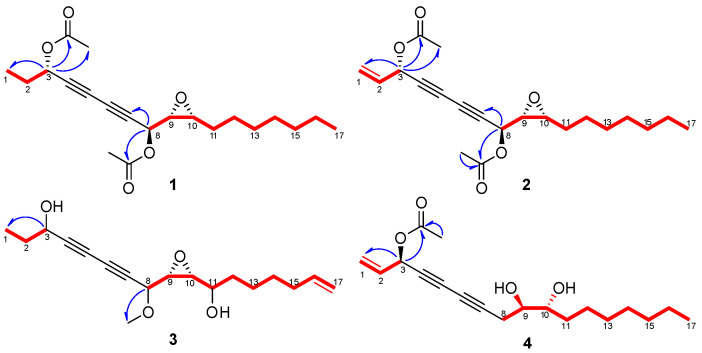
Key ^1^H-^1^H COSY (▬) and ^1^H-^13^C HMBC (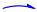) correlations among compounds **1**–**4**.

Compound **1** was isolated as yellow oil. The chemical formula was determined to be C_21_H_30_O_5_ by measuring HR-DART-MS (*m/z* 380.23495 [M + NH_4_]^+^; calcd for C_21_H_34_NO_5_, 380.24370) (Supplementary Materials Figure S1). The IR spectroscopy exhibited absorption bands at 1380, 1746, and 2250 cm^−1^, indicating that **1** has a conjugated triple bond and an acetyl group. The ^1^H NMR spectrum of **1** suggested the presence of two doublet methyl protons [*δ*_H_ 0.88 (3H, t, *J* = 7.0 Hz, H-17), 1.01 (3H, t, *J* = 7.5 Hz, H-1)] and two singlet methyl signals [*δ*_H_ 2.08 (3H, s, 3-OCOCH_3_), 2.11 (3H, s, 8-OCOCH_3_] (Table 1; Figure S2). Moreover, four oxygenated methine protons at *δ*_H_ 3.02 (1H, td, *J* = 6.0, 4.0 Hz, H-10), 3.21 (1H, dd, *J* = 7.5, 4.0 Hz, H-9), 5.23 (1H, dd, *J* = 7.5, 0.5 Hz, H-8), and 5.33 (1H, td, *J* = 6.5, 0.5 Hz, H-3) and aliphatic methylene groups at *δ*_H_ 1.25 (2H, m, H-14), 1.27 (2H, m, H-15), 1.28 (2H, m, H-16), 1.32 (2H, m, H-13), 1.39 (2H, m, H-12), and 1.57 (2H, m, H-11) were identified. Using the HSQC NMR spectrum of **1**, four methyl [*δ*c 9.5 (C-1), 14.3 (C-17), 20.9 (8-OCOCH_3_), 21.1 (3-OCOCH_3_)], seven methylene [*δ*c 22.8 (C-16), 26.7 (C-12), 27.8 (C-11), 28.0 (C-2), 29.4 (C-13), 29.5 (C-14), 32.0 (C-15)], four oxygenated methine [*δ*c 56.6 (C-9), 58.2 (C-10), 61.9 (C-8), 65.3 (C-3)], four quatenery carbon [*δ*c 69.3 (C-6), 70.8 (C-5), 74.4 (C-4), 77.7 (C-7)], and two carbonyl signals [*δ*c 169.1 (8-OCOCH_3_), 170.0 (3-OCOCH_3_)] were observed (Table 1; Figures S3 and S4). The COSY NMR spectrum revealed correlations between H-1 and H-2/H-3 and between H-8 and H-9/H-10, implying that the methyl, methylene, and oxygenated methine signals are connected (Figure 2 and Figure S5). The HMBC NMR spectrum displayed correlations between H-3 and 3-OCOCH_3_/3-OCOCH_3_ and between H-8 and C-7/8-OCOCH_3_, suggesting that the two acetoxy groups are located at C-3 and C-8 (Figure 2 and Figure S6).

**Figure 3:**
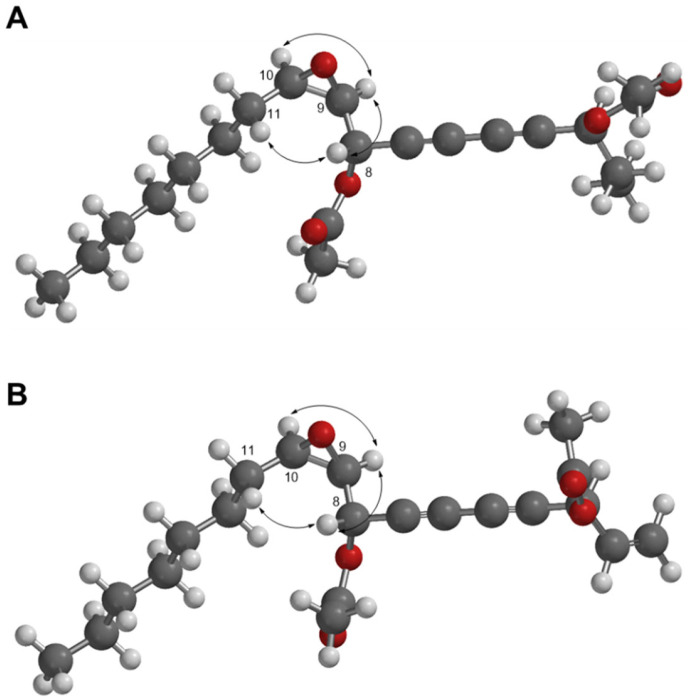
Key NOESY correlations (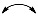) between compounds **1** and **2** (**A**,**B**). The 3D structures of **1** and **2** were modeled by Spartan′ 18.

The relative configuration of **1** was determined by considering the ^3^*J*_HH_ coupling constants and NOESY NMR data. Consistent correlations between H-9 with H-10 and the coupling constants for ^3^*J*_H9-H10_ (4.0 Hz) agreed well with the *cis* configuration [12]. Additionally, the NOESY correlation between H-8 and H-11/H-9 and a large coupling constant for ^3^*J*_H8-H9_ (7.5 Hz) indicated the *erythro* conformation for C-8 and C-9 (Figure 3 and Figure S7) [13,14]. Except for two extra acetyl groups, the structure of **1** was comparable to that of the previously reported (+)-oploxyne A [14,15]. The specific rotation value (−21.4) of **1** was different from that of (+)-oploxyne A. Therefore, the specific rotation value of **1** was calculated based on quantum mechanics using the HF/3-21G* basis set at wavelength 589 nm because sodium D-line was used in the experiments [15]. Four probable absolute configurations of **1** (3*R*/8*S*/9*R*/10*R*, 3*S*/8*S*/9*R*/10*R*, 3*R*/8*R*/9*S*/10*S*, and 3*S*/8*R*/9*S*/10*S*) were modeled and compared with the experimental data of **1**. The calculation result was −12.11 (Table S1), suggesting that the absolute configuration of **1** was 3*S*/8*S*/9*R*/10*R*. Therefore, the chemical structure of **1** was identified as (3*S*,8*S*,9*R*,10*R*)-(−)-heptadeca-9,10-epoxy-4,6-diyne-3,8-diyl diacetate, named ginsenoyne O. 

Compound **2** was isolated as yellow oil. The molecular formula of **2** was identified as C_21_H_28_O_5_ by HR-DART-MS (*m/z* 378.22787 [M + NH_4_]^+^; calcd for C_21_H_32_NO_5_, 378.22805) (Figure S8). The IR spectrum showed terminal double bond moiety at 1478 cm^−1^, acetyl moiety at 1676 cm^−1^, and triple bond of diyne moiety at 2255 cm^−1^. The ^1^H and DEPT NMR spectra of **2** were similar to **1**, except for the presence of exomethylene signals [*δ*_H_ 5.33 (1H, d, *J* = 10.0 Hz, H-1a), 5.52 (1H, d, *J* = 16.5 Hz, H-1b)/*δ*c 120.1 (C-1) and olefinic signals [*δ*_H_ 5.83 (1H, ddd, *J* = 15.5, 10.0, 6.5 Hz, H-2)/*δ*c 131.8 (C-2)] (Table 1; Figures S9 and S10). The above data indicated that **2** was an analog of **1**. The COSY and HMBC experiments led to the determination of the positions for all functional groups in **2** (Figure 2, Figure S11, and Figure S12). The *J* value analysis (*J*_H9-H10_ = 4.0 Hz/*J*_H8-H9_ = 7.5 Hz) and the NOESY correlations suggested that **2** had the same relative configuration as **1** (Table 1; Figure 3 and Figure S13). The absolute configuration of **2** was determined with the same method used for **1** (Table S1). Therefore, the structure of **2** was identified as (3*S*,8*S*,9*R*,10*R*)-(−)-heptadeca-1-en-9,10-epoxy-4,6-diyne-3,8-diyl diacetate, named ginsenoyne P.

Compound **3** was isolated as yellow oil, and its chemical formula was identified as C_18_H_26_O_4_ by HR-DART-MS (Figure S14). Using the MS, IR, and UV data, it was expected that **3** would have a similar structure to **1** and **2**. The ^1^H NMR spectrum exhibited an olefinic signal at *δ*_H_ 5.76 (H-16), five oxygenated methine protons [*δ*_H_ 2.90 (H-10), 3.19 (H-9), 3.40 (H-11), 4.01 (H-8), and 4.36 (H-3)], exomethylene protons at *δ*_H_ 4.90 (H-17a) and 4.96 (H-17b), a methyl proton at *δ*_H_ 0.99 (H-1), and one methoxy signal at *δ*_H_ 3.41 (8-OCH_3_) (Table 1; Figure S15). The ^13^C and HSQC NMR spectra presented one olefinic carbon at *δ*_C_ 114.7 (C-16), five oxygenated *sp*^3^ carbons [*δ*_C_ 57.0 (C-10), 59.4 (C-9), 64.3 (C-3), and 70.9 (C-8)], four *sp* carbons [*δ*_C_ 68.8 (C-7), 69.2 (C-6), 72.4 (C-5), and 81.2 (C-4)], one methyl signal at *δ*_C_ 9.5 (C-1), and one methoxy signal at *δ*_C_ 57.1 (8-OCH_3_) (Table 1; Figures S16 and S17). The connections from C-1 to C-3 and from C-8 to C-17 in **3** were confimed using an analysis of the COSY data (Figure S18). The position of the methoxy group was determined to be C-8 using an analysis of the HMBC spectrum (Figure 3 and Figure S19). The stereochemistry of the epoxide ring was determined to be *cis* configuration using the *J* value analysis (^3^*J*_H9-H10_ = 4.5 Hz). Although the NOESY spectrum exhibited correlations between H-8/H-11 and H-9/H-10 (Figure S20), the relative energies of all conformers exhibited approximate values in the conformer search for **3**. As a highly oxygenated derivative with scarce amount, we could not determine the absolute configuration of **3**. Thus, the structure of **3** was identified as (−)-haptadeca-9,10-epoxy-8-methoxy-4,6-diyne-3,11-diol, named ginsenoyne Q. 

Compound **4** was isolated as yellow oil, and its molecular formula was identified as C_19_H_28_O_4_ by HR-DART-MS (Figure S21). The IR spectrum of **4** showed terminal double bond moiety at 1226 cm^−1^, acetyl moiety at 1746 cm^−1^, and a triple bond band of diyne moiety at 2307 cm^−1^. Careful analysis of the ^1^H NMR spectrum revealed that the epoxide ring observed in **1**–**3** was absent in **4** (Figure S22). The ^1^H and ^13^C NMR spectra of **4** were almost identical with those of (3*R*,9*R*,10*R*)-panaxytriol (**11**), which is a major polyacetylene from ginseng and which was also isolated in this study, except for the presence of an acetyl group in **4** (Table 2, Figures S22–S27). The position of the acetyl group in **4** was determined to be C-3 using an analysis of the HMBC spectrum (Figure 2 and Figure S26), implicating that **4** is a different compound from a known 10-acetyl panaxytriol [16]. The coupling constant of H-9 (^3^*J*_H9-H10_ = 5.5 Hz) in **4** was the same as that of **11**, implicating that they have the same relative configurations at C-9 and C-10. To determine the absolute configuration, deacetylation of **4** was conducted to yield **4a** (Figure S28). The specific rotation value of **4a** (−24.2) was similar to those of **11** (−19.5) and (3*R*,9*R*,10*R*)-panaxytriol in the literature (−18.6) [17], indicating that they have the same absolute configuration. Therefore, the structure of **4** was elucidated as (3*R*,9*R*,10*R*)-(−)-3-acetoxy-9,10-dihydroxyheptadeca-1-en-4,6-diyne, named 3-acetyl panaxytriol.

The structures of the known compounds were identified as ginsenoyne E (**5**) [18], acetylpanaxydol (**6**) [19], ginsenoyne G (**7**) [20], ginsenoyne H (**8**) [20], (3*R*,9*R*,10*S*)-panaxydol (**9**) [21], ginsenoyne F (**10**) [20], (3*R*,9*R*,10*R*)-panaxytriol (**11**) [17], ginsenoyne C (**12**) [22], panaxynol (**13**) [23], falcarinone (**14**) [24], panaquinquecol 4 (**15**) [25], 1-methoxy-(9*R*,10*S*)-epoxyheptadecan-4,6-diyn-3-one (**16**) [26], (8*E*)-1,8-heptadecadiene-4,6-diyne-3,10-diol (**17**) [21], (3*R*,8*E*,10*S*)-8-heptadecene-4,6-diyne-3,10-diol (**18**) [27], and ginsenoyne K (**19**) [21] by comparing their NMR spectroscopic data with those from previously reported studies.

### 2.2. The Cytotoxicity of C_17_ Polyacetylenes against Human Ovarian Cancer Cells

**Figure 4:**
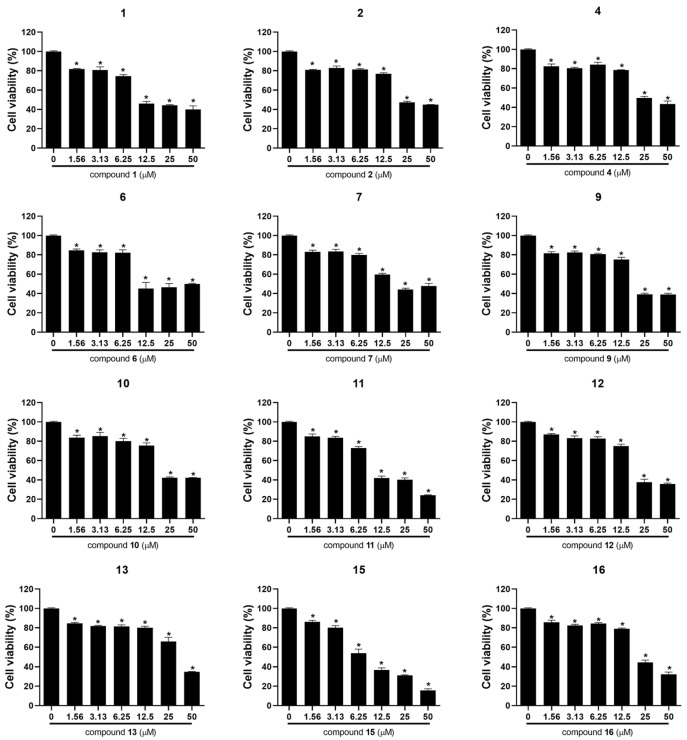
The effects of compounds **1**−**2**, **4**, **6**−**7**, **9**−**13**, and **15**−**16** on cell viability in epithelial ovarian cancer cells A2780. The results are presented as mean standard ± SD and were processed by using a one-way ANOVA. * *p* < 0.05 in comparison to control (0 μM).

**Figure 5:**
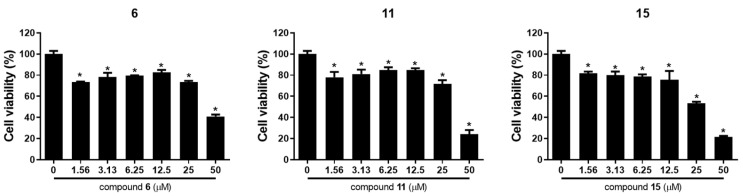
The effects of compounds **6**, **11**, and **15** on cell viability in epithelial ovarian cancer cells SKOV3. The results are presented as mean standard ± SD and were processed by using a one-way ANOVA. * *p* < 0.05 in comparison to control (0 µM).

To evaluate the effect of the C_17_ polyacetylenes isolated in this study on the viability of ovarian cancer cells, MTT assay was performed on A2780 and SKOV3 human ovarian cancer cells. As shown in Figure 4 and Table 3, ginsenoyne O (**1**), ginsenoyne P (**2**), 3-acetyl panaxytriol (**4**), acetylpanaxydol (**6**), ginsenoyne G (**7**), (3*R*,9*R*,10*S*)-panaxydol (**9**), ginsenoyne F (**10**), (3*R*,9*R*,10*R*)-panaxytriol (**11**), ginsenoyne C (**12**), panaxynol (**13**), panaquinquecol 4 (**15**), and 1-methoxy-(9*R*,10*S*)-epoxyheptadecan-4,6-diyn-3-one (**16**) significantly inhibited the viability of A2780 cells in a dose-dependent manner. However, only acetylpanaxydol (**6**), (3*R*,9*R*,10*R*)-panaxytriol (**11**), and panaquinquecol 4 (**15**) exhibited remarkable cytotoxic activities against SKOV3 cells with IC_50_ values below 50 μM (Table 3 and Figure 5). Among these, panaquinquecol 4 (**15**) showed the most potent cytotoxic activity against A2780 and SKOV3 cells with IC_50_ values of 7.60 ± 1.33 and 27.53 ± 1.22 μM, respectively. 

Among the isolates, acetylpanaxydol (**6**), (3*R*,9*R*,10*R*)-panaxytriol (**11**), and panaquinquecol 4 (**15**) showed remarkable cytotoxic activities against both ovarian cancer cells (A2780 and SKOV3). To investigate compounds **6**, **11**, and **15** as potential anti-cancer agents by regulating the tumor microenvironments, the cytotoxicity of these compounds was evaluated against RAW264.7 macrophages, the normal cell lines (Table S2). Compounds **11** and **15** showed less cytotoxicity against RAW264.7 (IC_50_ values of 20.03 ± 0.53 and 18.61 ± 0.75 μM, respectively).

## 3. Discussion

Polyacetylenes, commonly found as secondary metabolites in plants, have one or more carbon-carbon triple bonds in their structure. However, because there are various secondary metabolic pathways involved in the biosynthesis of polyacetylene, these compounds are sometimes highly oxidized and may contain a variety of substituents. Ginseng (*Panax* spp.) is one of the most well-known plants that contains a significant amount of polyacetylene in addition to ginsenosides [28]. For many years, researchers have become more interested in discovering novel ginsenosides as well as polyacetylenes [29]. In this study, four newly reported C17 polyacetylenes—ginsenoynes O, P, and Q (**1**–**3**), and 3-acetyl panaxytriol (**4**)—were isolated from the fresh roots of *P*. *ginseng*. Although the presence of panaquinquecol 4 (**15**) and (3*R*,8*E*,10*S*)-8-heptadecene-4,6-diyne-3,10-diol (**18**) was reported in *Panax quinquefolium* and *Oplopanax horridus*, it was reported for the first time in *P*. *ginseng* in this study. 

Epithelial ovarian cancer is classified into several types based on histologic and molecular characteristic [30]. Therefore, high-throughput screening with a variety of epithelial ovarian cancer cell lines is needed for the discovery of selective inhibitors. Polyacetylenes are some of the most significant phytochemicals that contribute to cytotoxic, anti-inflammatory, and potential anti-cancer properties [11,31]. Increasing scientific evidence has shown that panaxdol (**9**) and (3*R*,9*R*,10*R*)-panaxytriol (**11**) are viable anti-cancer drug candidates against epithelial ovarian cancer cells (A2780) as major compounds in *P*. *ginseng* [32,33]. However, this is the first report that acetylpanaxydol (**6**), ginsenoyne G (**7**), ginsenoyne F (**10**), ginsenoyne C (**12**), panaquinquecol 4 (**15**), and 1-methoxy-(9*R*,10*S*)-epoxyheptadecan-4,6-diyn-3-one (**16**) have cytotoxic effects on ovarian cancer cells.

In this study, the polyacetylenes were more effective against the A2780 cells compared to SKOV3 cells. These results indicated that the p53 pathway is likely associated with compound-induced cell death, and SKOV3 cells without p53 activity seem to be less responsive against the compounds. Nevertheless, panaquinquecol 4 (**15**) exhibited significant cytotoxicity in both A2780 and SKOV3 cells (7.60 ± 1.33 and 27.53 ± 1.22 μM, respectively). It has been reported that panaquinquecol 4 (**15**), which was first isolated from *P*. *quinquefolium*, has strong cytotoxicity against human leukemia cells (L1210), with an IC_50_ value 20 times lower than panaquinquecol 5, the C_14_ polyacetylene [25]. However, the cytotoxic effects of panaquinquecol 4 (**15**) against other carcinoma cell lines and their mechanisms are not yet fully understood. Therefore, further investigation into this compound is required.

## 4. Materials and Methods

### 4.1. General Experimental Procedure

Thin-layer chromatography (TLC) analysis was performed using RP-18 F254S and silica gel 60 F254 plates (Merck, Kenilworth, NJ, USA). Column chromatography (CC) using Sephadex LH-20 (Amersham Pharmacia Biotech, Buckinghamshire, United Kingdom), Diaion HP-20 (Mitsubishi, Tokyo, Japan), and silica gel (70-230 and 230–400 mesh ASTM, Merck) was implemented to isolate compounds. A medium-pressure liquid chromatography (MPLC) purification system was used with pre-packed cartridges, Redi Sep-C18 and Redi Sep-Silica (Teledyne Isco, Lincoln, NE, USA). Preparative HPLC was performed using the Waters purification system (1525 pump, PDA 1996 detector, Waters, Milford, MA, USA) with Gemini 5 μm NX-C18 110A column (250 × 21.2 mm i.d., 5.0 μm, Phenomenex, Torrance, CA, USA) and Discovery 5 μm C18 column (250 × 10.0 mm i.d., 5.0 μm, Supelco, Bellefonte, PA, USA).

The following spectroscopic measurements were obtained for the purified compounds: nuclear magnetic resonance (NMR) spectra using a 500 MHz (JEOL, Tokyo, Japan) and an 800 MHz NMR spectrometer (Bruker, MA, USA); UV spectra using an Optizen pop apparatus (Mecasys, Daejeon, Korea); and optical rotations using a 10 mm microcell on a Jasco P-2000 polarimeter (JASCO, Tokyo, Japan). High-resolution mass spectra (HR-MS) were obtained using an AccuTOF^®^ single-reflectron time-of-flight mass spectrometer (JEOL Ltd., Tokyo, Japan) equipped with direct analysis in real-time (DART) ion source (IonSense, Saugus, MA, USA). 

### 4.2. Plant Materials

The fresh roots of *Panax ginseng* C.A. Meyer (Araliaceae) were purchased from a local market (Chungcheongnam-do, Korea) and authenticated by Prof. Dae Sik Jang. A voucher specimen (PAGI-2019) of the raw material was deposited in the Laboratory of Natural Product Medicine, College of Pharmacy, Kyung Hee University, Seoul, Republic of Korea.

### 4.3. Extraction and Isolation of Polyacetylenes from P. ginseng

The sliced raw material (40.53 kg) was extracted with 80 L of 70% acetone for 2 weeks at room temperature, and the solvent was evaporated at 40 °C. The above extraction procedure was repeated once. The 70% acetone extract was then suspended in H_2_O (6.4 L) and extracted with EtOAc (6.4 L × 3) to yield EtOAc- (85.59 g) and water-soluble extracts (772.07 g). The EtOAC-soluble extract was fractionated using silica gel CC (70–230 mesh; *ф* 6.5 × 48.0 cm) with a gradient system of *n*-hexane-EtOAc (95:5 to 80:20, *v*/*v*) to yield 24 fractions (E1 ~ E24). E3 (4.08 g) was separated using Sephadex LH-20 CC (*ф* 4.5 × 55.8 cm) with methylene chloride (MC) to yield nine subfractions (E3-1 ~ E3-9). E3-2 was separated using MPLC with a Redi Sep-Silica cartridge (80 g, *n*-hexane-EtOAc = 100:0 to 75:25 *v*/*v*) to yield six subfractions (E3-2-1 ~ E3-2-6). E3-2-5 was separated using MPLC with a Redi Sep-C18 gold cartridge (5.5 g, THF-H_2_O = 70:30 to 75:25 *v*/*v*) to afford five subfractions (E3-2-5-1 ~ E3-2-5-5). Compounds **1** (1.0 mg) and **2** (1.0 mg) were purified using semi-prep HPLC with a Discovery column from E3-2-5-1. E3-6 was subjected to a silica gel CC (*ф* 5.0 × 34.8 cm) with *n*-hexane-EtOAc (from 10:0 to 70:30 *v*/*v*) to yield eight subfractions (E3-6-1 ~ E3-6-8). Panaxynol (**13**, 658.5 mg), falcarinone (**14**, 67.1 mg), and ginsenoyne K (**19**, 15.6 mg) were purified using MPLC with a Redi Sep-C18 cartridge (130 g, acetonitrile-H_2_O = 65:35 to 90:10 *v*/*v*) from E3-6-3. E5 (11.74 g) was subjected to Sephadex LH-20 CC (*ф* 4.5 × 59.0 cm) with MC to yield five subfractions (E5-1 ~ E5-5). Subfraction E5-4 was separated using silica gel CC (*ф* 5.0 × 33.3 cm) with *n*-hexane-EtOAc (from 95:5 to 80:20 *v*/*v*) to yield ginsenoyne E (**5**, 48.4 mg) and four subfractions (E5-4-1 ~ E5-4-4). Panaquinquecol 4 (**15**, 4.4 mg) was purified using prep HPLC with a Gemini column from E5-4-3. E5-4-4 was chromatographed using a Redi Sep-Silica cartridge (80 g, *n*-hexane-EtOAc = 100:0 to 70:30 *v*/*v*) to afford panaxydol (**9**, 430.1 mg) and 1-methoxy-(9*R*,10*S*)-epoxyheptadecan-4,6-diyn-3-one (**16**, 9.1 mg). E2 (2.76 g) was separated using Sephadex LH-20 CC (*ф* 4.8 × 64.3 cm) with MC to produce six subfractions (E2-1 ~ E2-6). E2-2 was separated using MPLC with a Redi Sep-Silica cartridge (24 g, MC-acetone = 80:20 to 60:40 *v*/*v*) to yield eight subfractions (E2-2-1 ~ E2-2-8). E2-2-5 was subjected to MPLC using a Redi Sep-C18 cartridge (26 g, acetonitrile-H_2_O = 65:35 to 95:5, *v*/*v*) to isolate acetylpanaxydol (**6**, 18.6 mg), ginsenoyne G (**7**, 8.6 mg), and ginsenoyne H (**8**, 1.0 mg). E2-2-6 produced ginsenoyne F (**10**, 4.1 mg) by using HPLC with a Gemini column. E14 (8.27 g) was separated into four subfractions (E14-1 ~ E14-4) using Sephadex LH-20 CC (*ф* 4.8 × 64.6 cm) with MC-MeOH = 50:50. Subfraction E14-3 was fractionated using a silica gel CC (*ф* 5.0 × 34.5 cm) with *n*-hexane-EtOAc = 60:40 to afford panaxytriol (**11**, 726.4 mg) and ginsenoyne C (**12**, 5.1 mg). Subfraction E10 (1.15 g) was chromatographed using Sephadex LH-20 CC (*ф* 4.8 × 64.6 cm) with MC-MeOH (70:30, *v*/*v*) to produce five subfractions (E10-1 ~ E10-5). E10-4 was separated using prep HPLC with a Gemini column to generate (8*E*)-1,8-heptadecadiene-4,6-diyne-3,10-diol (**17**, 4.5 mg) and (3*R*,8*E*,10*S*)-heptadec-8-ene-4,6-diyne-3,10-diol (**18**, 0.7 mg). E12 (436.6 mg) was fractionated using Sephadex LH-20 CC (*ф* 4.8 × 71.0 cm) with MC-MeOH (70:30, *v*/*v*) to produce seven subfractions (E12-1 ~ E12-7). E12-3 was chromatographed over a Redi Sep-C18 cartridge (26 g, MeOH-H_2_O = 60:40 to 80:20 *v*/*v*) to afford compounds **3** (1.5 mg) and **4** (4.2 mg).

#### 4.3.1. Ginsenoyne O (**1**)

Yellow oil; [α]_D_^22^ −21.43; UV (Acetonitrile) λ_max_ (log ε) 233 nm (3.05), 245 nm (2.88), 258 nm (2.70); IR (neat) ν_max_ 1380, 1746, 2250; DART-MS *m/z* 380.23495 [M + H]^+^ (calcd for C_21_H_31_O_5_, 380.24370); ^1^H and ^13^C NMR data. See Table 1.

#### 4.3.2. Ginsenoyne P (**2**)

Yellow oil; [α]_D_^22^ −5.96; UV (Acetonitrile) λ_max_ (log ε) 233 nm (2.99), 246 nm (2.69), 260 nm (2.50); IR (neat) ν_max_ 1478, 1676, 2255; DART-MS *m/z* 378.22787 [M + NH_4_]^+^ (calcd for C_21_H_32_NO_5_, 378.22805); ^1^H and ^13^C NMR data. See Table 1.

#### 4.3.3. Ginsenoyne Q (**3**)

Yellow oil; [α]_D_^22^ −4.90; UV (Acetonitrile) λ_max_ (log ε) 224 nm (3.04), 245 nm (2.83), 258 nm (2.72); IR (neat) ν_max_ 2944, 3003, 3469; DART-MS *m/z* 324.21625 [M + NH_4_]^+^ (calcd for C_18_H_30_NO_4_, 324.21748); ^1^H and ^13^C NMR data. See Table 1.

#### 4.3.4. 3-Acetyl Panaxytriol (**4**)

Yellow oil; [α]_D_^22^ 33.03; UV (Acetonitrile) λ_max_ (log ε) 222 nm (2.90), 233 nm (2.95), 245 nm (2.82); IR (neat) ν_max_ 1226, 1746, 2307; DART-MS *m/z* 338.23114 [M + NH_4_]^+^ (calcd for C_19_H_32_NO_4_, 338.23313); ^1^H and ^13^C NMR data. See Table 2. 

### 4.4. Computational Chemistry

At the Hartree-Fock level of theory, ab initio calculations for **1** were performed. Using Gaussian 16 software (version G16 C.01; Wallingford, CT, USA) and modified versions of the reported methods, all geometries and conformers of **1** were optimized using an HF/3-21* level of theory [15]. The cumulative Boltzmann distribution (up to 75%, Table S1) was used among generated conformers to select the conformers of **1** for calculating the specific rotation. Specific rotations of single conformers at 589 nm were calculated using a CPCM/HF/3-21* level of theory (chloroform). The final specific rotation value of **1** was calculated using the relative Boltzmann weighting of conformers.

### 4.5. Deacetylation of Compound **4**

Acid hydrolysis was performed to confirm the absolute configuration of **4** using the modified procedures previously described [34]. Compound **4** (1.0 mg) and 1N HCl (0.01 mL) in MeOH (1.0 mL) were stirred at room temperature for 24 h. After the reaction, it was partitioned with EtOAc and water to obtain a product. The EtOAc fraction was purified directly using HPLC under a gradient system [A: water, B: methanol, 70% B, 35 min].

### 4.6. Cell Culture and MTT Assay

A2780 and SKOV3 human ovarian cancer cell lines and RAW264.7 macrophages were obtained from American Type Culture Collection (ATCC). The cell culture and MTT assay were performed using the method described previously [35,36].

## 5. Conclusions

Four new C_17_ polyacetylenes, ginsenoyne O (**1**), ginsenoyne P (**2**), ginsenoyne Q (**3**), and 3-acetyl panaxytriol (**4**), along with fifteen known ones (**5**-**19**), were isolated from the fresh roots of *P*. *ginseng*; moreover, the chemical structures of **1**-**4** were elucidated using 1D- and 2D- NMR spectroscopy and specific rotation calculation. Among the 19 polyacetylenes*,* acetylpanaxydol (**6**), (3*R*,9*R*,10*R*)-panaxytriol (**11**), and panaquinquecol 4 (**15**) exhibited a potent cytotoxic effect against both A2780 and SKOV3 human ovarian cells. This finding warrants further investigation into the anti-tumor activity of these C_17_ polyacetylenes in human ovarian cancer.

## Figures and Tables

**Table 1:** ^1^H and ^13^C NMR spectroscopic data of compounds **1**−**3** (*δ* in ppm, chloroform-*d*).

Position	1		2		3	
	*δ*_H_ (multi *J* in Hz) *^a^*	*δ*_C_ *^b^*	*δ*_H_ (multi *J* in Hz) *^a^*	*δ*_C_ *^c^*	*δ*_H_ (multi *J* in Hz) *^a^*	*δ*_C_ *^d^*
1	1.01 (7.5)	9.5	5.33 d (10.0) 5.52 d (16.5)	120.1	0.99 t (7.5)	9.5
2	1.80 qd (7.5, 6.5)	28.0	5.83 ddd (15.5, 10.0, 6.5)	131.8	1.79 d (6.0)	30.8
3	5.33 td (6.5, 0.5)	65.3	5.88 td (6.5, 1.0)	64.3	4.36 dd (12.0, 6.0)	64.3
4		74.4		74.9		81.2
5		70.8		70.4		72.4
6		69.3		70.5		69.2
7		77.7		75.2		68.8
8	5.23 dd (7.5, 0.5)	61.9	5.22 dd (7.5, 0.5)	61.7	4.01 d (7.0)	70.9
9	3.21 dd (7.5, 4.0)	56.6	3.19 dd (7.5, 4.0)	56.3	3.19 dd (7.5, 4.5)	59.4
10	3.02 td (6.0, 4.0)	58.2	3.00 td (6.0, 3.5)	58.0	2.90 dd (8.5, 4.0)	57.0
11	1.57 m	27.8	1.54 m	26.5	3.40 m	70.0
12	1.39 *^e^* m	26.7	1.23 *^e^*	29.7	1.67 m	34.9
13	1.32 *^e^* m	29.4	1.29 *^e^*	29.3	1.42 *^e^*	24.8
14	1.25 *^e^* m	29.5	1.23 *^e^*	31.7	1.42 *^e^*	29.1
15	1.27 *^e^* m	32.0	1.26 *^e^*	22.6	2.03 dd (12.5, 5.5)	33.9
16	1.28 m	22.8	1.46	27.6	5.76 ddd (17.0, 10.5, 6.5)	114.7
17a	0.88 t (7.0)	14.3	0.86 t (6.5)	14.1	4.90 ddd (10.0, 2.0, 1.5)	139.0
17b					4.96 ddd (12.0, 2.0, 1.5)	
3-OCOCH_3_	2.08 s	21.1	2.08 s	20.7		
3-OCOCH_3_	-	169.1		168.9		
8-OCOCH_3_	2.11 s	20.9 170.0	2.09 s	20.7 169.4		
8-OCOCH_3_	-					
OMe					3.41 s	57.1

*^a^* The signals were observed at 500 MHz. *^b^* The signals were observed at 200 MHz. *^c^* The signals were confirmed by HMBC. *^d^* The signals were observed at 125 MHz. *^e^* The signals overlapped.

**Table 2:** ^1^H and ^13^C NMR spectroscopic data of compound **4** and panaxytriol (**11**) (*δ* in ppm, chloroform-*d*, 500 and 125 MHz).

Position	4	panaxytriol (11)		
	*δ*_H_ (multi *J* in Hz)	*δ*_C_	*δ*_H_ (multi *J* in Hz)	*δ*_C_
1a 1b	5.34 ddd (9.5, 1.0, 1.0) 5.54 ddd (16.5, 1.0, 1.0)	119.8	5.23 ddd (10.0, 1.0, 1.0) 5.45 ddd (17.0, 1.0, 1.0)	117.4
2	5.86 ddd (16.5, 10.0, 5.0)	132.3	5.92 ddd (17.0, 10.0, 5.0)	136.2
3	5.89 dq (5.5, 1.0)	64.7	4.90 br t (5.0)	63.6
4		71.4		75.0
5		71.6		71.1
6		66.6		66.6
7		78.6		78.3
8a 8b	2.55 ddd (17.5, 5.5, 1.0) 2.60 ddd (17.0, 6.0, 1.0)	25.1	2.55 ddd (17.5, 6.0, 1.0) 2.59 ddd (17.5, 6.0, 1.0)	25.8
9	3.64 dt (5.5, 5.5)	72.3	3.63 dt (5.5, 5.5)	72.4
10	3.59 dt (5.5)	73.2	3.57 m	73.3
11	1.50 *^a^* m	34.0	1.48 *^a^* m	33.7
12a 12b	1.35 *^a^* m 1.46 *^a^* m	25.7	1.35 *^a^* m 1.45 *^a^* m	25.1
13	1.27 *^a^* m	29.7	1.22–1.31	29.8
14	1.27 *^a^* m	29.4		29.4
15	1.25 *^a^* m	32.0		32.0
16	1.30 *^a^* m	22.9		22.9
17	0.88 t (7.0)	14.3	0.87 t (7.0)	14.2
3-OCOCH_3_	2.10 s	21.1		
3-OCOCH_3_		169.8		
9-OH	2.00 br s		2.34 d (5.0)	
10-OH	2.40 br s		2.74 d (5.5)	

*^a^* The signals overlapped.

**Table 3:** The cytotoxicity of compounds **1**−**19** isolated from *P. ginseng* in A2780 and SKOV3 human ovarian cancer cells.

Compound	IC_50_ (µM) *^a^*		Compound	IC_50_ (µM) *^a^*	
	A2780	SKOV3		A2780	SKOV3
**1**	11.64 ± 0.44	>50	**11**	10.90 ± 0.25	36.38 ± 1.95
**2**	23.86 ± 0.49	>50	**12**	20.88 ± 0.69	>50
**3**	>50	>50	**13**	37.63 ± 1.48	>50
**4**	25.80 ± 2.24	>50	**14**	>50	>50
**5**	>50	>50	**15**	7.60 ± 1.33	27.53 ± 1.22
**6**	11.80 ± 0.81	43.10 ± 1.15	**16**	22.96 ± 0.76	>50
**7**	20.25 ± 0.28	>50	**17**	>50	>50
**8**	>50	>50	**18**	>50	>50
**9**	21.25 ± 0.12	>50	**19**	>50	>50
**10**	22.09 ± 0.12	>50			

*^a^* IC_50_ value is the concentration that results in a 50% reduction in cell number when compared to control cultures. Cisplatin used as a positive control. The IC_50_ value of cisplatin against A2780 and SKOV3 cells were 20.87 ± 0.94 and 27.65 ± 0.83 µM, respectively.

## Data Availability

Not applicable.

## Supplementary Materials

The following supporting information can be downloaded at: https://www.mdpi.com/article/10.3390/molecules27207027/s1, Figure S1: HR-DART-MS spectrum of compound **1**; Figure S2: ^1^H NMR spectrum of compound **1** (500 MHz, chloroform-*d*); Figure S3: ^13^C NMR spectrum of compound **1** (200 MHz, chloroform-*d*); Figure S4: ^1^H ^13^C HSQC spectrum of compound **1**; Figure S5: ^1^H ^1^H COSY spectrum of compound **1**; Figure S6: ^1^H ^13^C HMBC spectrum of compound **1**; Figure S7: ^1^H ^1^H NOESY spectrum of compound **1**; Figure S8: HR-DART-MS spectrum of compound **2**; Figure S9: ^1^H NMR spectrum of compound **2** (500 MHz, chloroform-*d*); Figure S10: DEPT NMR spectrum of compound **2** (125 MHz, chloroform-*d*); Figure S11: ^1^H ^1^H COSY spectrum of compound **2**; Figure S12: ^1^H ^13^C HMBC spectrum of compound **2**; Figure S13: ^1^H ^1^H NOESY spectrum of compound **2**; Figure S14: HR-DART-MS spectrum of compound **3**; Figure S15: ^1^H NMR spectrum of compound **3** (500 MHz, chloroform-*d*); Figure S16: ^13^C NMR spectrum of compound **3** (125 MHz, chloroform-*d*); Figure S17: ^1^H ^13^C HSQC spectrum of compound **3**; Figure S18: ^1^H ^1^H COSY spectrum of compound **3**; Figure S19: ^1^H ^13^C HMBC spectrum of compound **3**; Figure S20: ^1^H ^1^H NOESY spectrum of compound **3**; Figure S21: HR-DART-MS spectrum of compound **4**; Figure S22: ^1^H NMR spectrum of compound **4** (500 MHz, chloroform-*d*); Figure S23: ^13^C NMR spectrum of compound **4** (125 MHz, chloroform-*d*); Figure S24: ^1^H ^13^C HSQC spectrum of compound **4**; Figure S25: ^1^H ^1^H COSY spectrum of compound **4**; Figure S26: ^1^H ^13^C HMBC spectrum of compound **4**; Figure S27: ^1^H ^1^H NOESY spectrum of compound **4**; Figure S28: ^1^H NMR spectra of compounds **11** and **4a**; Table S1. Experimental and calculated specific rotation data of **1** and **2**; Table S2. The cytotoxicity of compounds **6**, **11**, and **15** isolated from *P*. *ginseng* in RAW264.7 macrophages.
