# Supplementary material for: Cytotoxic Properties of C17 Polyacetylenes from the Fresh Roots of Panax ginseng on Human Epithelial Ovarian Cancer Cells
Source: Molecules. 2022 Oct 18;27(20):7027. doi: 10.3390/molecules27207027 (PMC9607587; doi:10.3390/molecules27207027)
Supplement: Supplementary file 1 [file molecules-27-07027-s001.zip › molecules-1965282-supplementary.pdf]

Supporting information

Contents

Figure S1. HR-DART-MS spectrum of compound **1**

Figure S2. ^{1}H NMR spectrum of compound **1** (500 MHz, chloroform-*d*)

Figure S3. ^{13}C NMR spectrum of compound **1** (200 MHz, chloroform-*d*)

Figure S4. ^{1}H ^{13}C HSQC spectrum of compound **1**

Figure S5. ^{1}H ^{1}H COSY spectrum of compound **1**

Figure S6. ^{1}H ^{13}C HMBC spectrum of compound **1**

Figure S7. ^{1}H ^{1}H NOESY spectrum of compound **1**

Figure S8. HR-DART-MS spectrum of compound **2**

Figure S9. ^{1}H NMR spectrum of compound **2** (500 MHz, chloroform-*d*)

Figure S10. DEPT NMR spectrum of compound **2** (125 MHz, chloroform-*d*)

Figure S11. ^{1}H ^{1}H COSY spectrum of compound **2**

Figure S12. ^{1}H ^{13}C HMBC spectrum of compound **2**

Figure S13. ^{1}H ^{1}H NOESY spectrum of compound **2**

Figure S14. HR-DART-MS spectrum of compound **3**

Figure S15. ^{1}H NMR spectrum of compound **3** (500 MHz, chloroform-*d*)

Figure S16. ^{13}C NMR spectrum of compound **3** (125 MHz, chloroform-*d*)

Figure S17. ^{1}H ^{13}C HSQC spectrum of compound **3**

Figure S18. ^{1}H ^{1}H COSY spectrum of compound **3**

Figure S19. ^{1}H ^{13}C HMBC spectrum of compound **3**

Figure S20. ^{1}H ^{1}H NOESY spectrum of compound **3**

Figure S21. HR-DART-MS spectrum of compound **4**

Figure S22. ^{1}H NMR spectrum of compound **4** (500 MHz, chloroform-*d*)

Figure S23. ^{13}C NMR spectrum of compound **4** (125 MHz, chloroform-*d*)

Figure S24. ^{1}H ^{13}C HSQC spectrum of compound **4**

Figure S25. ^{1}H ^{1}H COSY spectrum of compound **4**

Figure S26. ^{1}H ^{13}C HMBC spectrum of compound **4**

Figure S27. ^{1}H ^{1}H NOESY spectrum of compound **4**

Figure S28. ^{1}H NMR spectra of compounds **11** and **4a**

Figure S29. The effects of compounds **1−2**, **4**, **6−7**, **9−13**, and **15−16** on cell viability in epithelial ovarian cancer cells A2780

Figure S30. The effects of compounds **6**, **11**, and **15** on cell viability in epithelial ovarian cancer cells SKOV3

Table S1. Experimental and calculated specific rotation data of **1** and **2**

Table S2. The cytotoxicity of compounds **6**, **11**, and **15** isolated from *P. ginseng* in RAW264.7 macrophages

Figure S1. HR-DART-MS spectrum of compound **1**

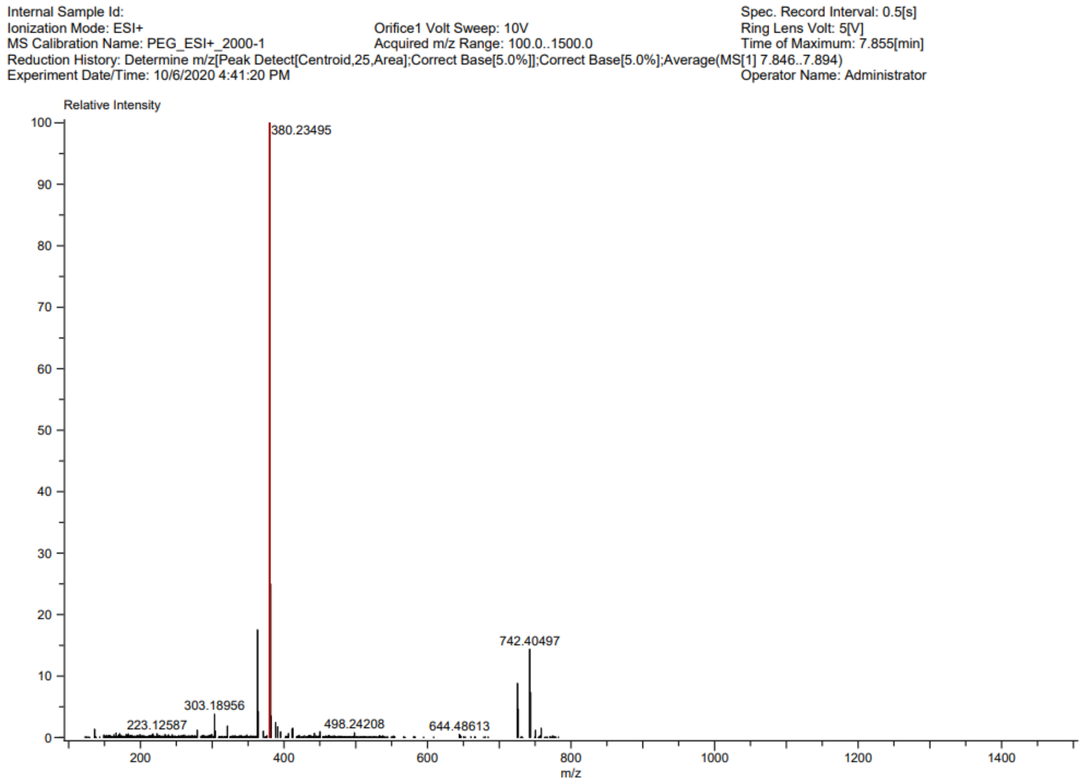

Figure S2. ^{1}H NMR spectrum of compound **1** (500 MHz, chloroform-*d*)

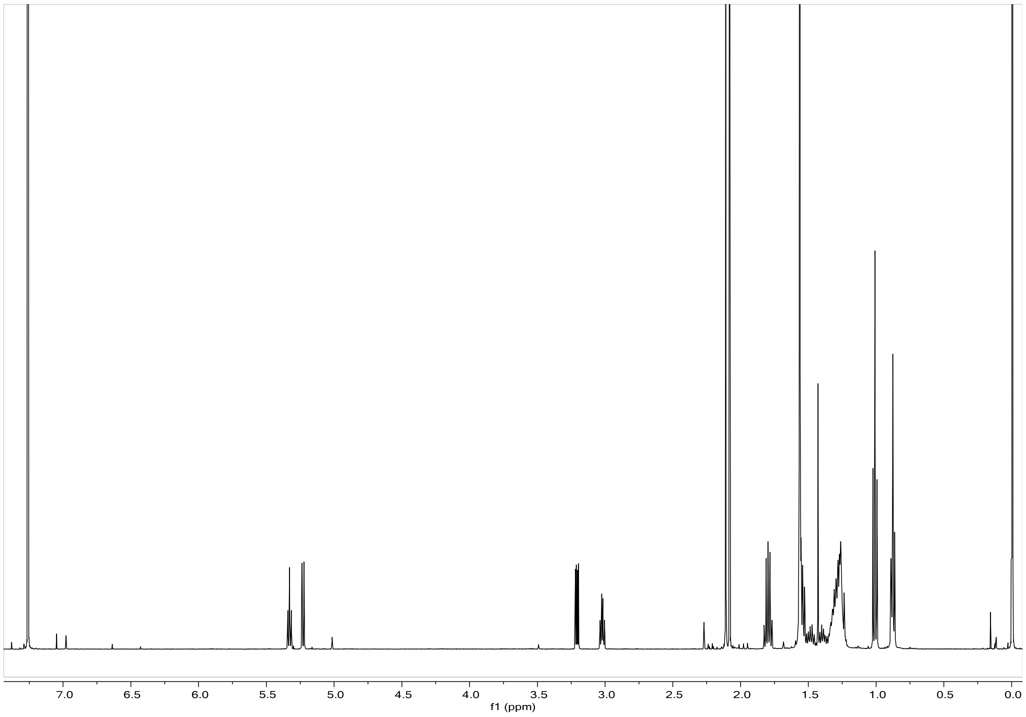

Figure S3. ^{13}C NMR spectrum of compound **1** (200 MHz, chloroform-*d*)

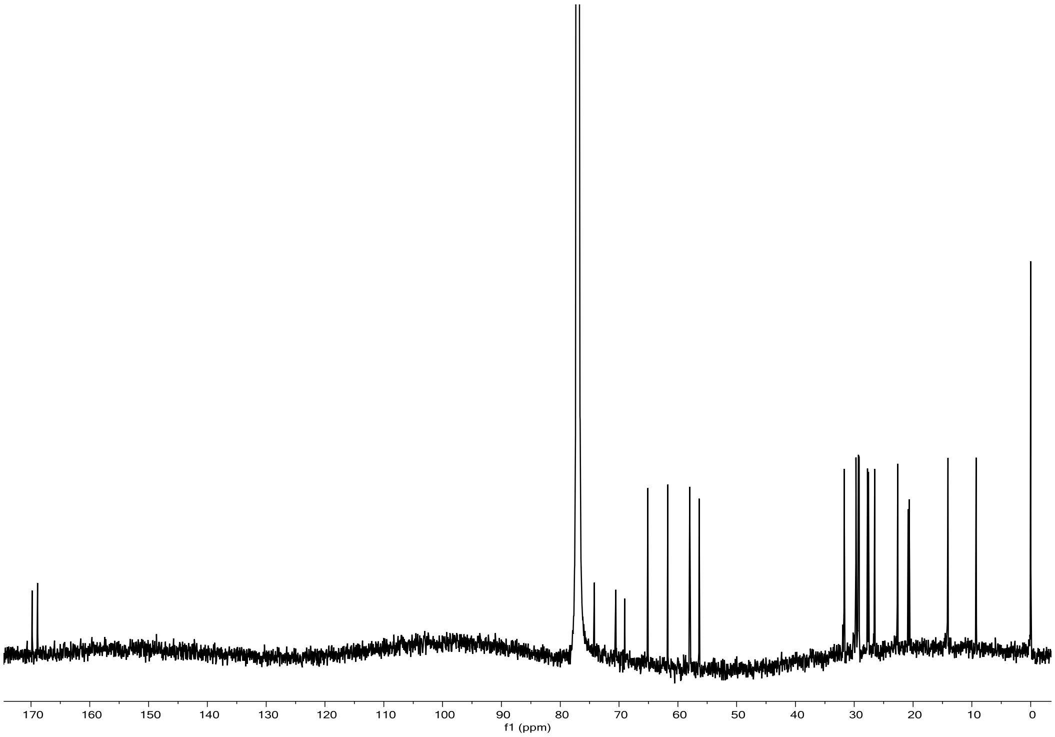

Figure S4 ^{1}H ^{13}C HSQC spectrum of compound **1**

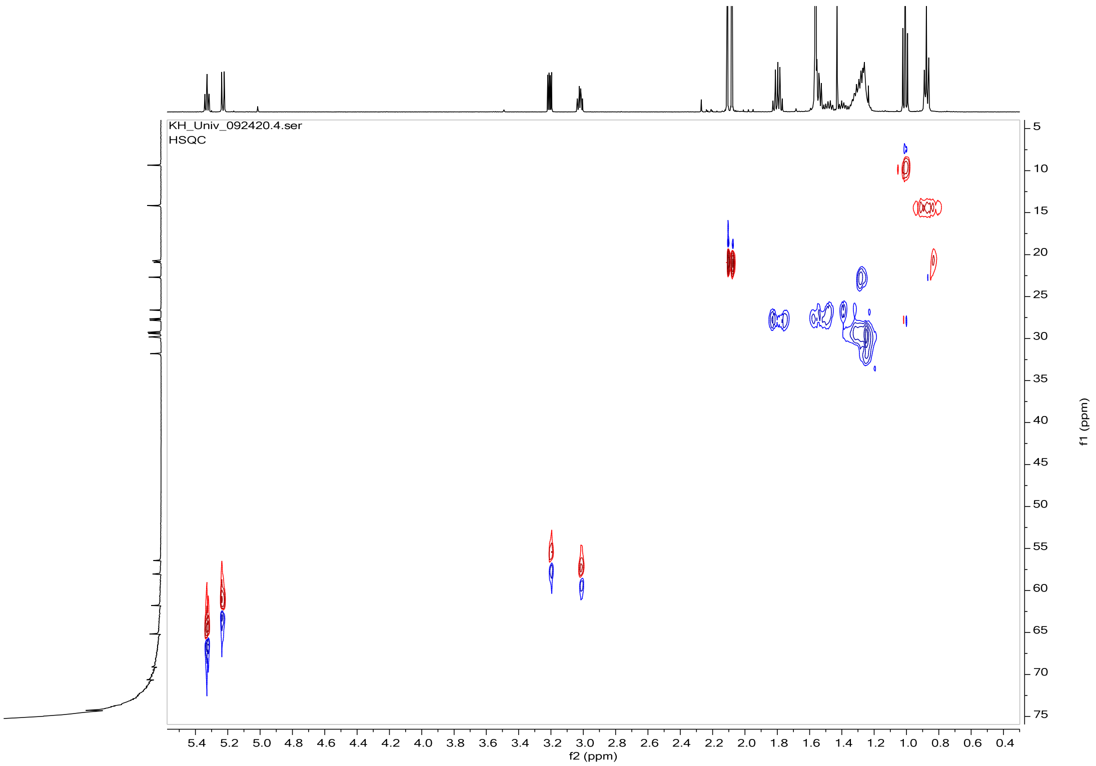

Figure S5. ^1H ^1H COSY spectrum of compound **1**

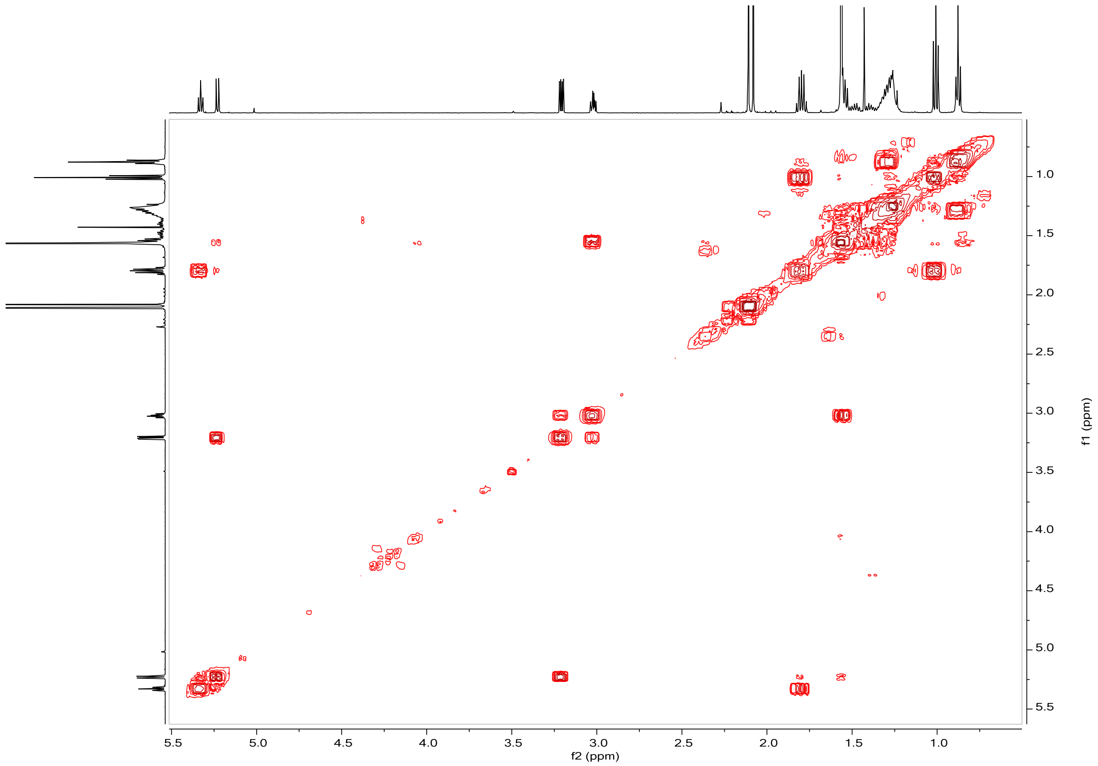

Figure S6. ^1H ^{13}C HMBC spectrum of compound **1**

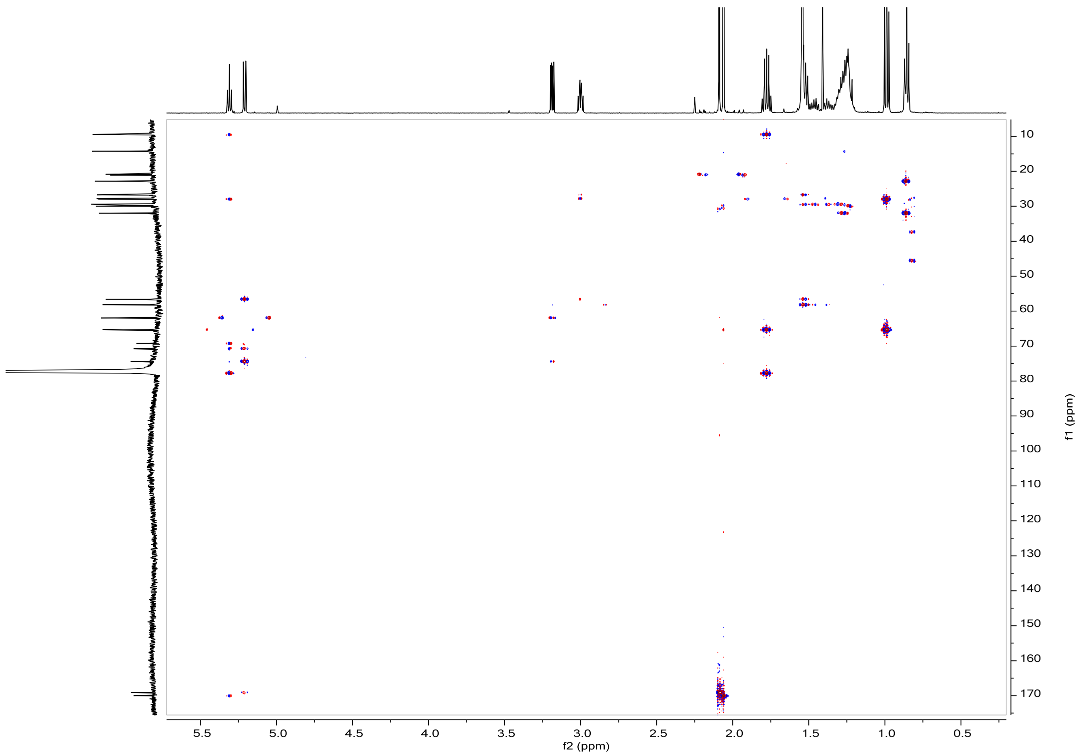

Figure S7. ^{1}H ^{1}H NOESY spectrum of compound **1**

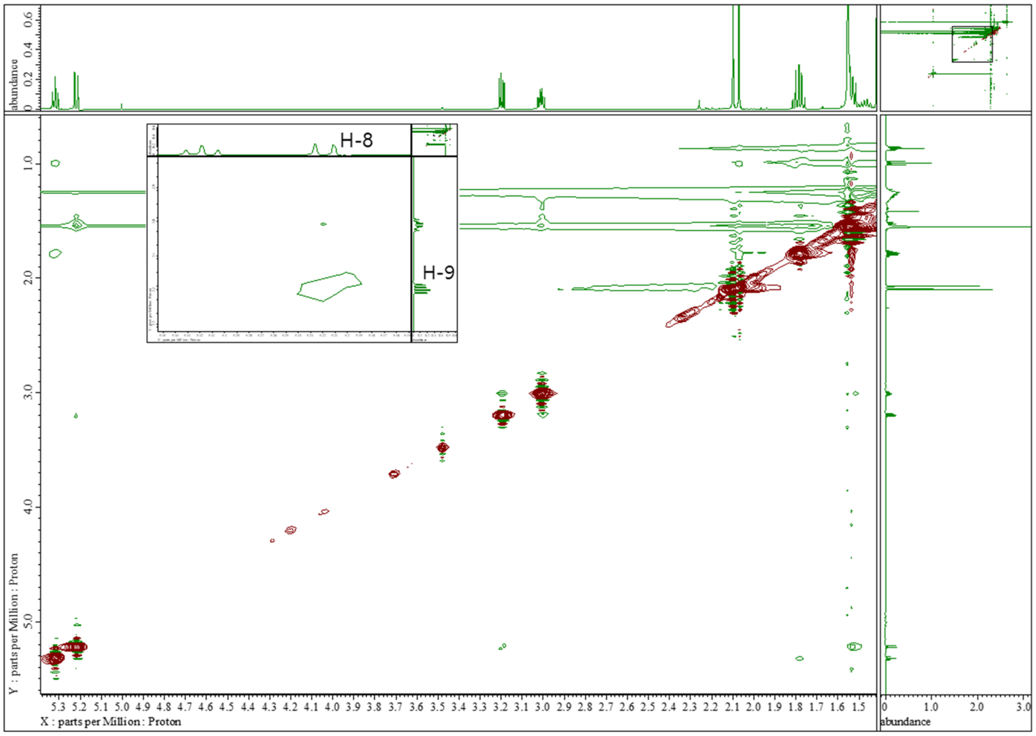

Figure S8. HR-DART-MS spectrum of compound **2**

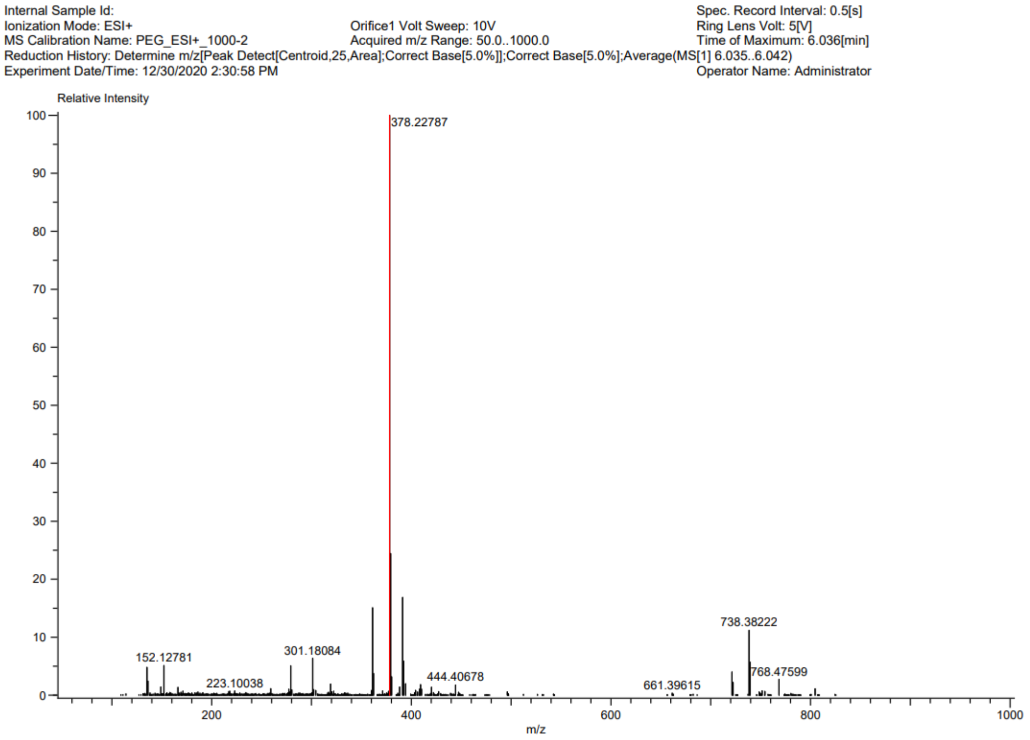

Figure S9. ^{1}H NMR spectrum of compound **2** (500 MHz, chloroform-*d*)

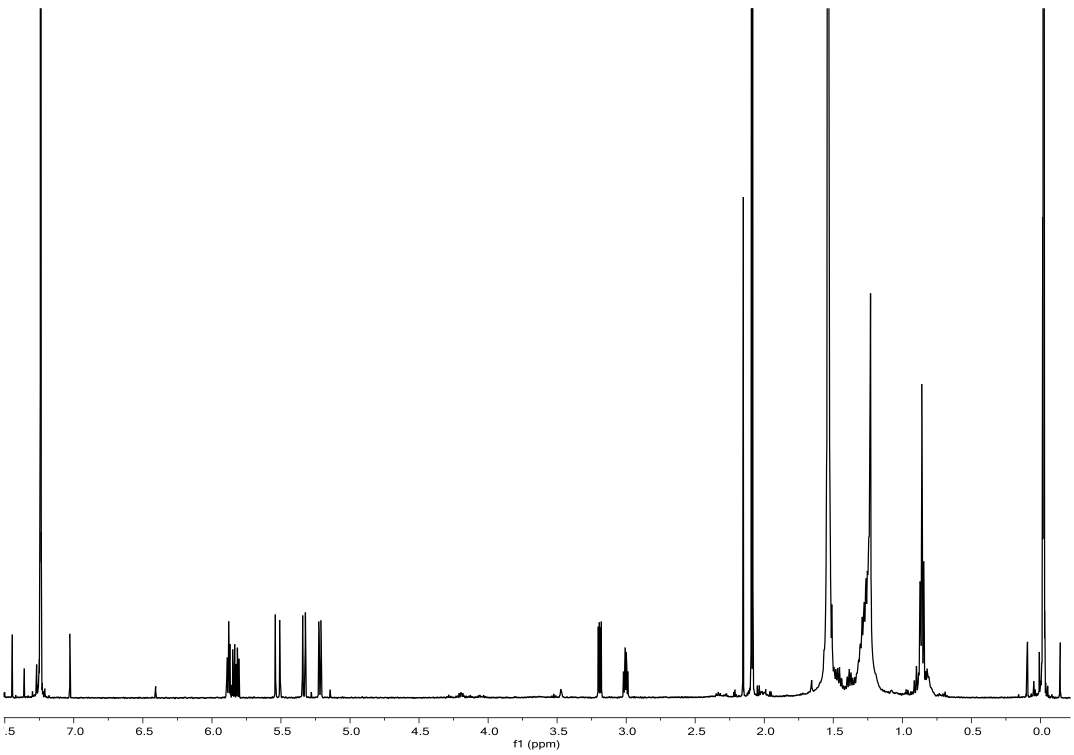

Figure S10. DEPT NMR spectrum of compound **2** (125 MHz, chloroform-*d*)

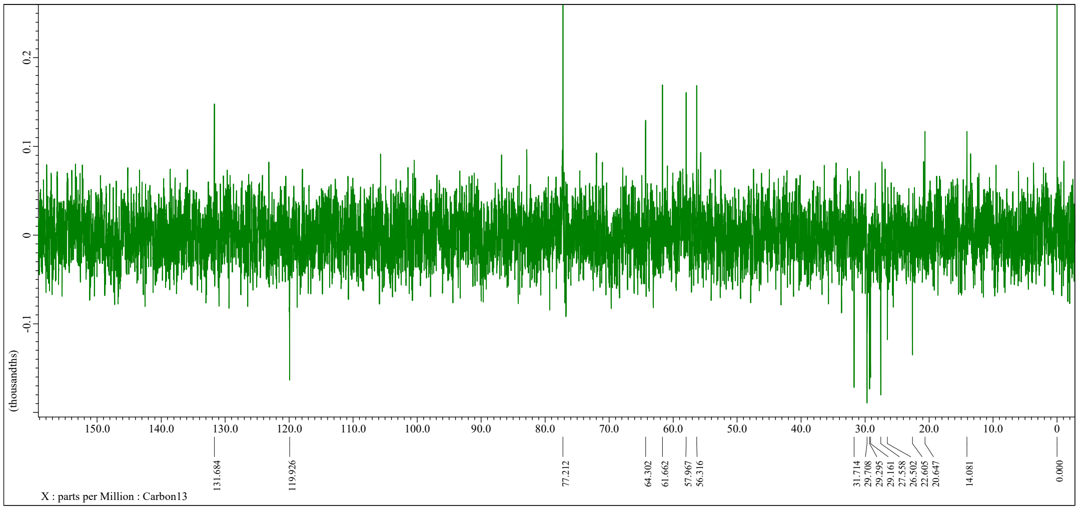

Figure S11. ^{1}H ^{1}H COSY spectrum of compound **2**

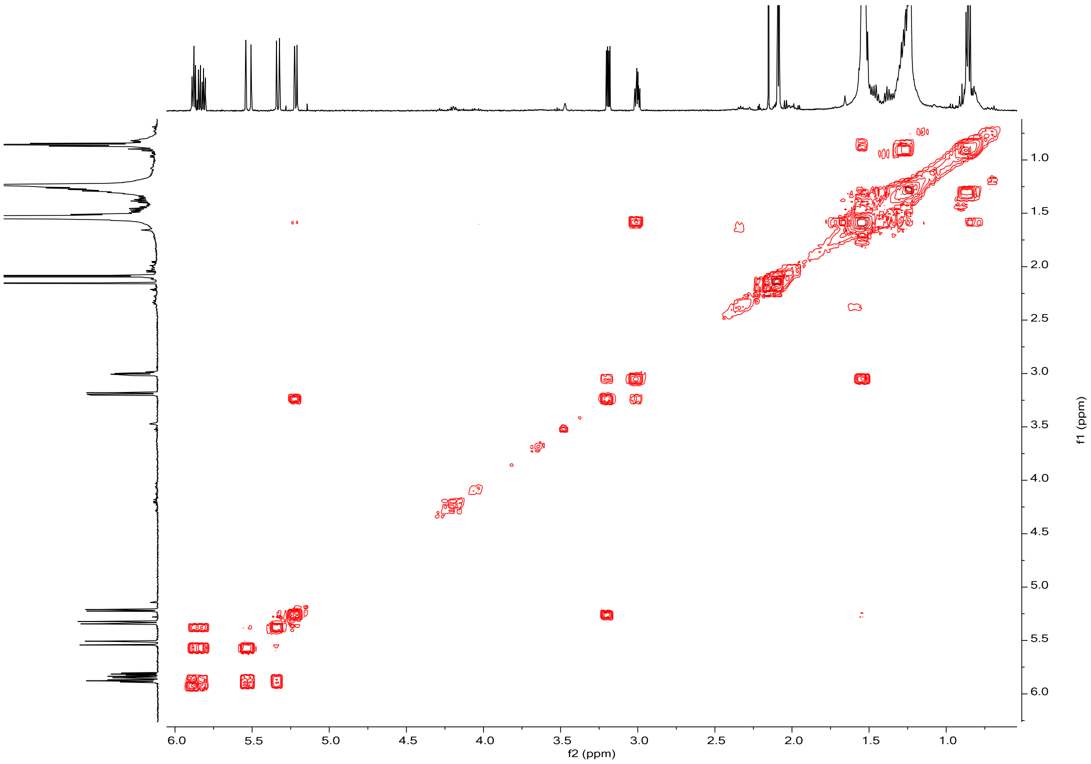

Figure S12. ^{1}H ^{13}C HMBC spectrum of compound **2**

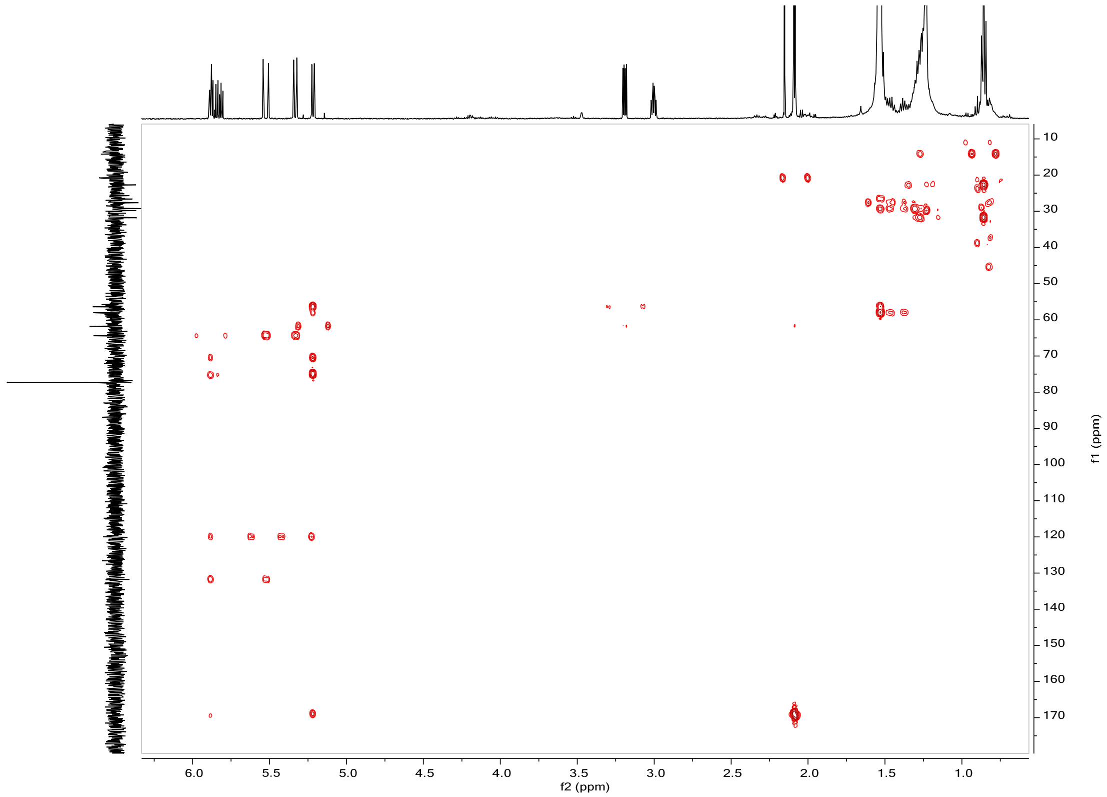

Figure S13. ^{1}H ^{1}H NOESY spectrum of compound **2**

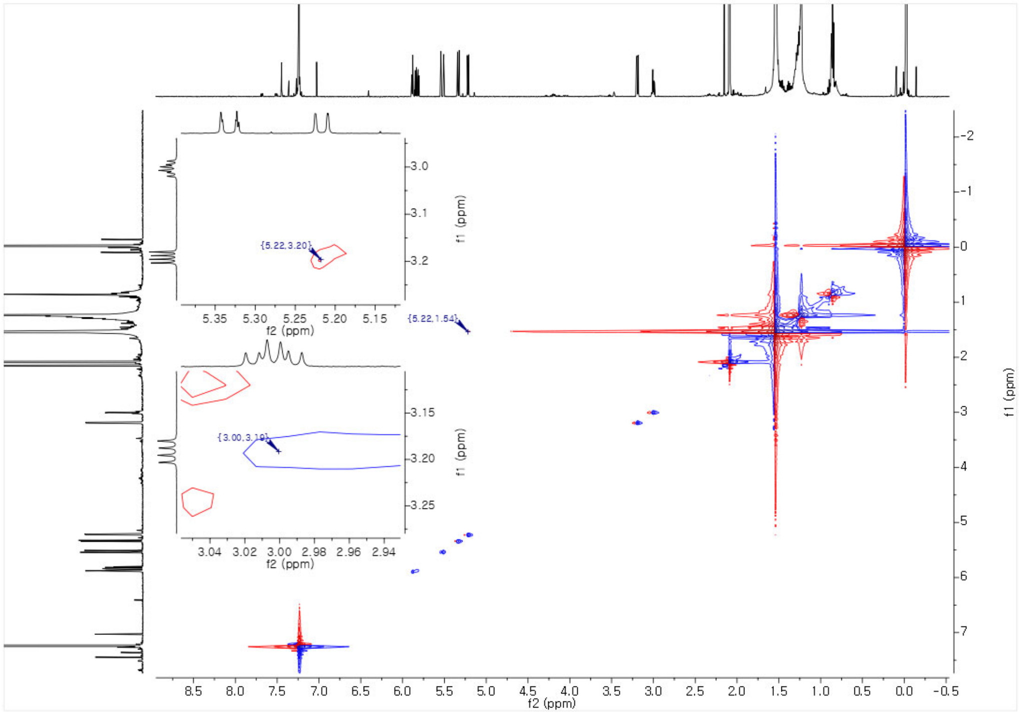

Figure S14. HR-DART-MS spectrum of compound **3**

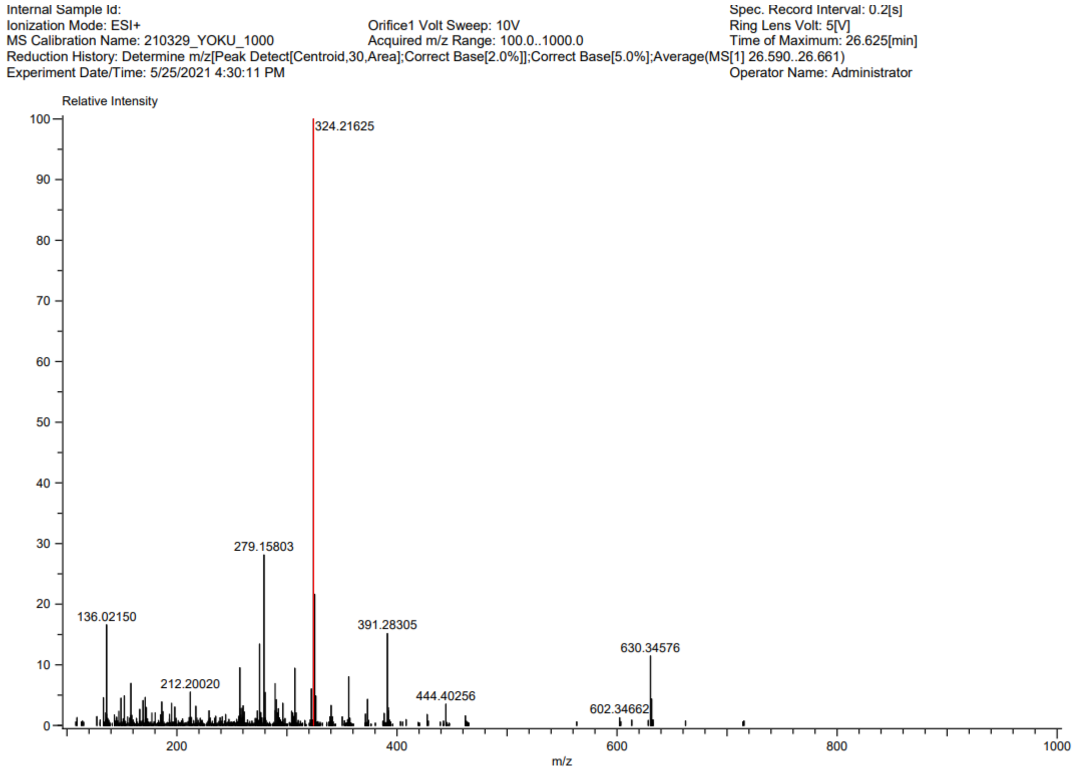

Figure S15. ^{1}H NMR spectrum of compound **3** (500 MHz, chloroform-*d*)

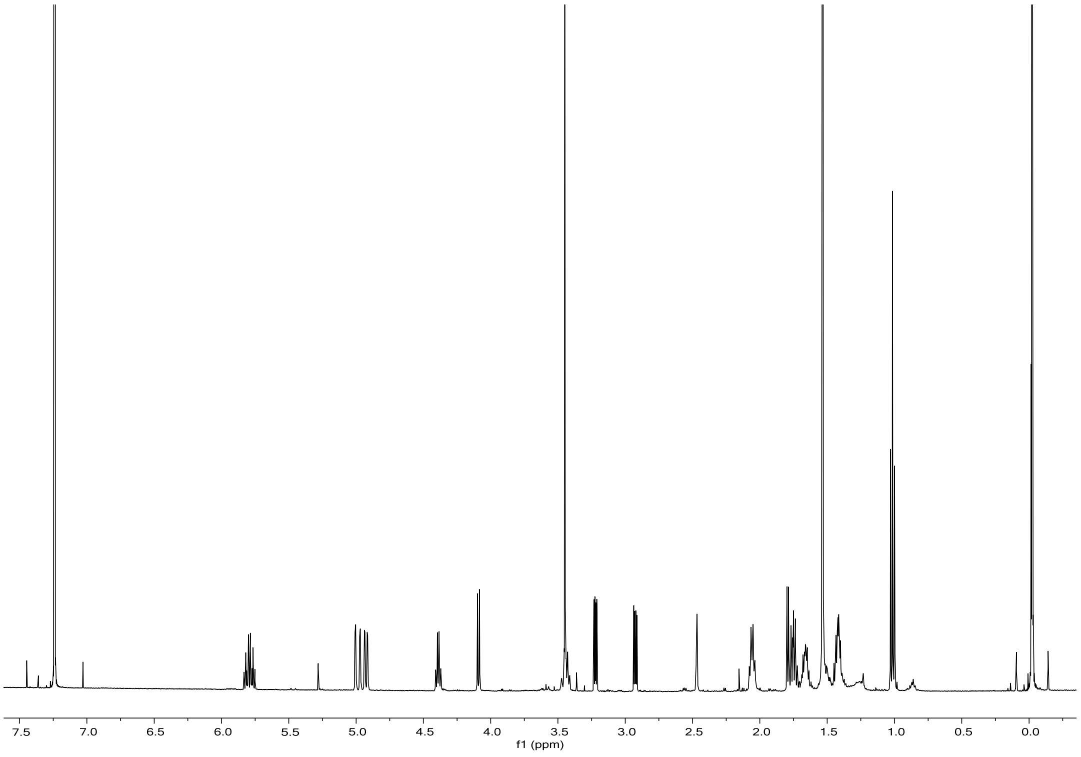

Figure S16. ^{13}C NMR spectrum of compound **3** (125 MHz, chloroform-*d*)

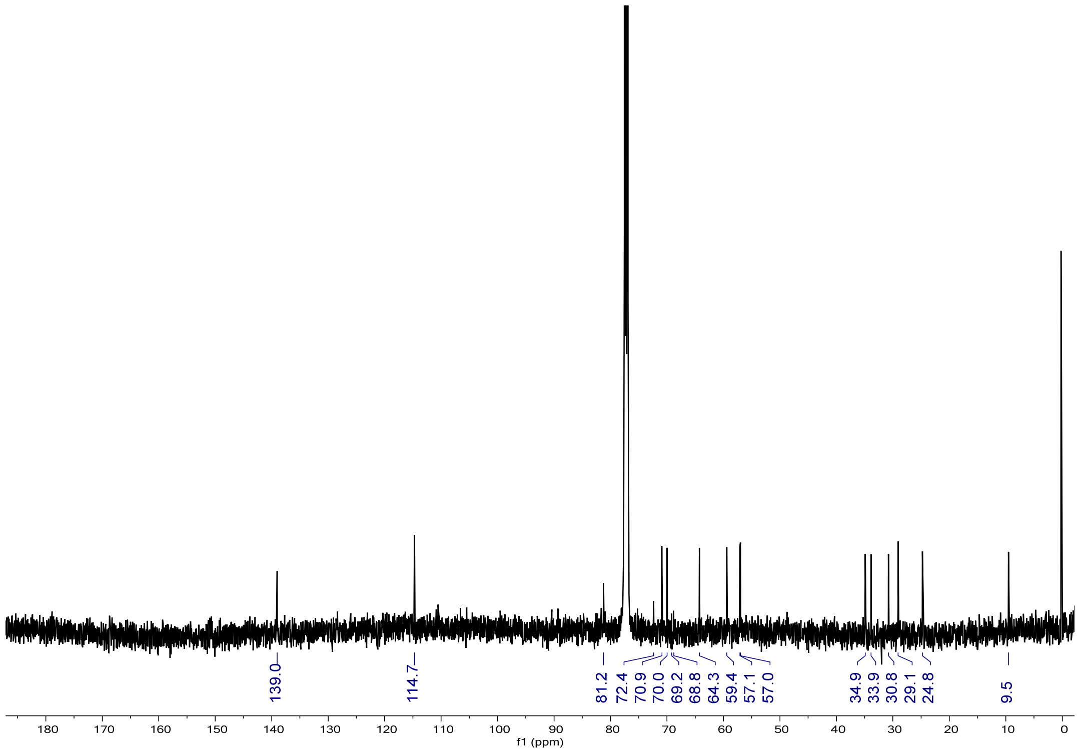

Figure S17. ^{1}H ^{13}C HSQC spectrum of compound **3**

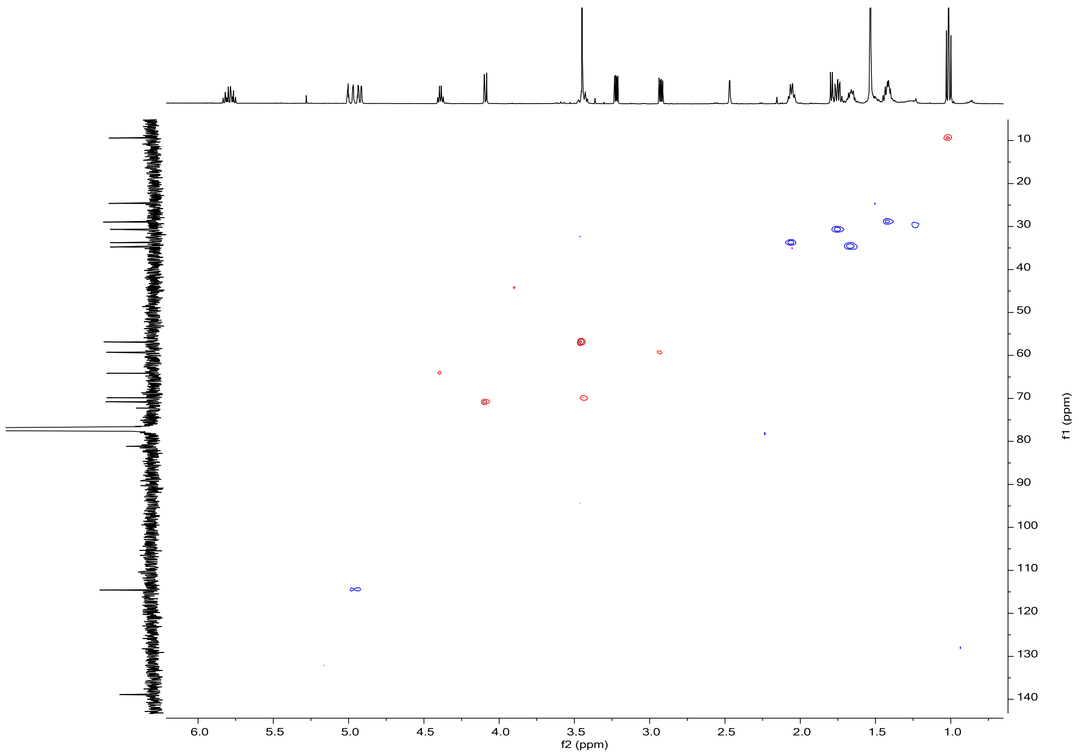

Figure S18. ^{1}H ^{1}H COSY spectrum of compound **3**

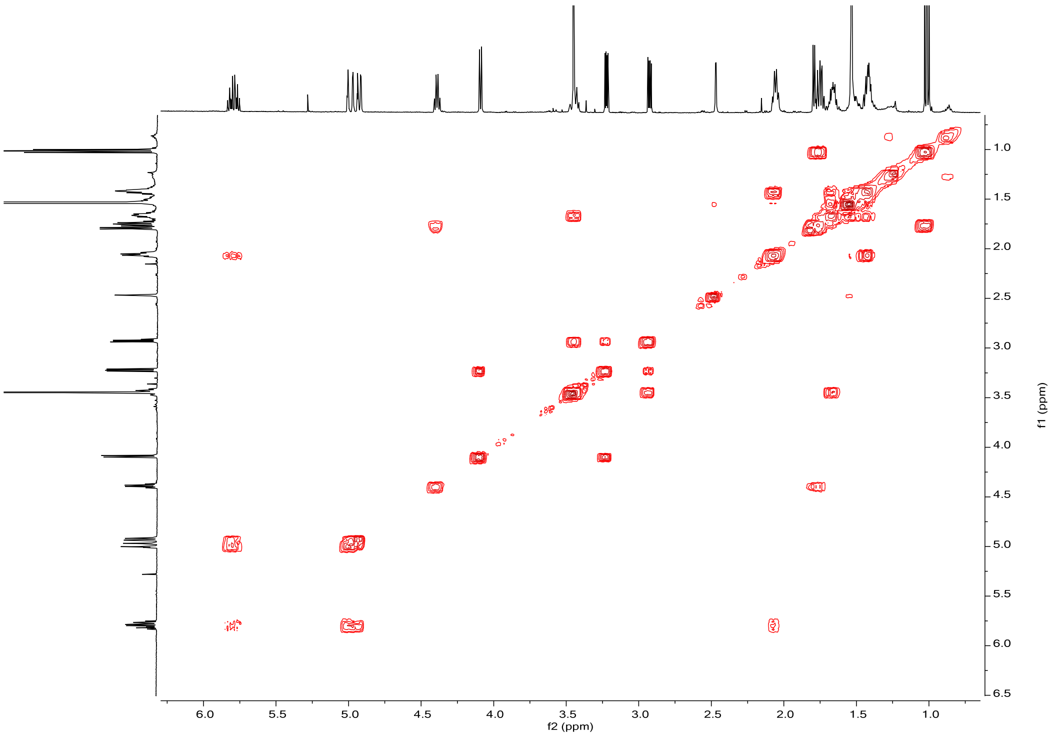

Figure S19. ^{1}H ^{13}C HMBC spectrum of compound **3**

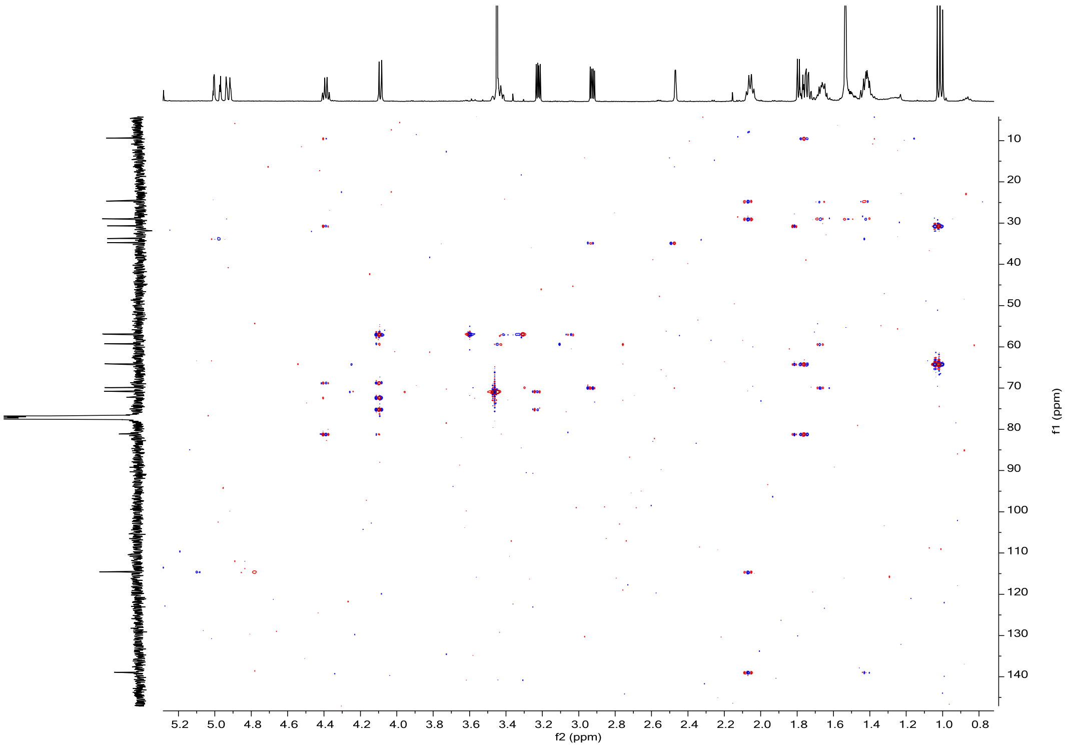

Figure S20. ^{1}H ^{1}H NOESY spectrum of compound **3**

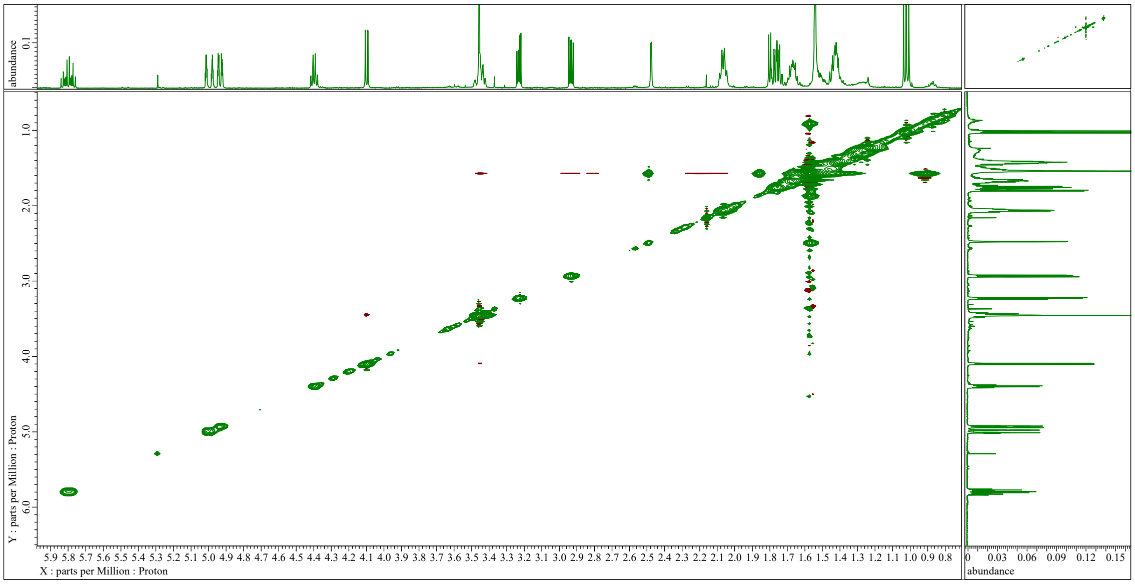

Figure S21. HR-DART-MS spectrum of compound **4**

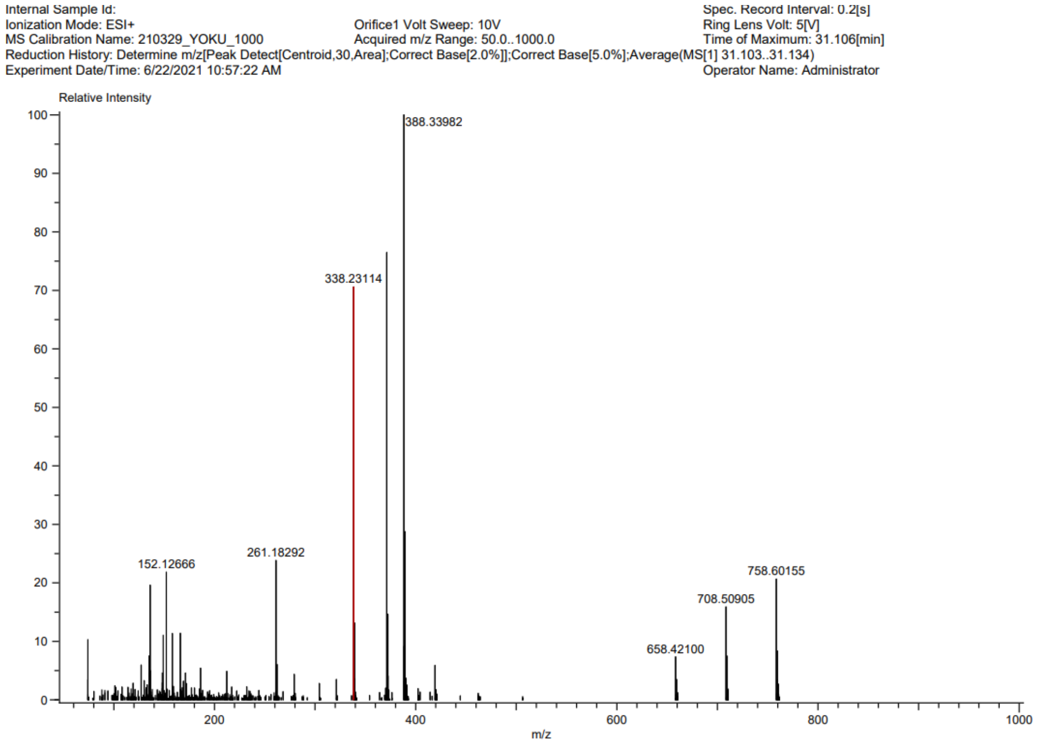

Figure S22. ^{1}H NMR spectrum of compound **4** (500 MHz, chloroform-*d*)

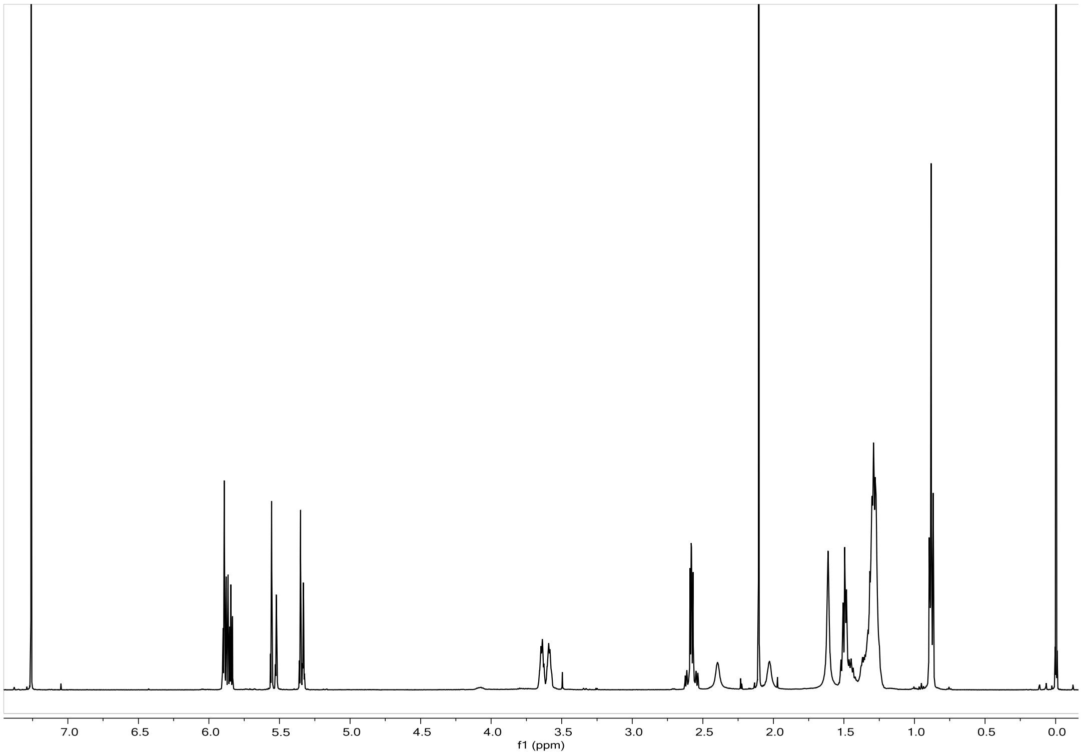

Figure S23. ^{13}C NMR spectrum of compound **4** (125 MHz, chloroform-*d*)

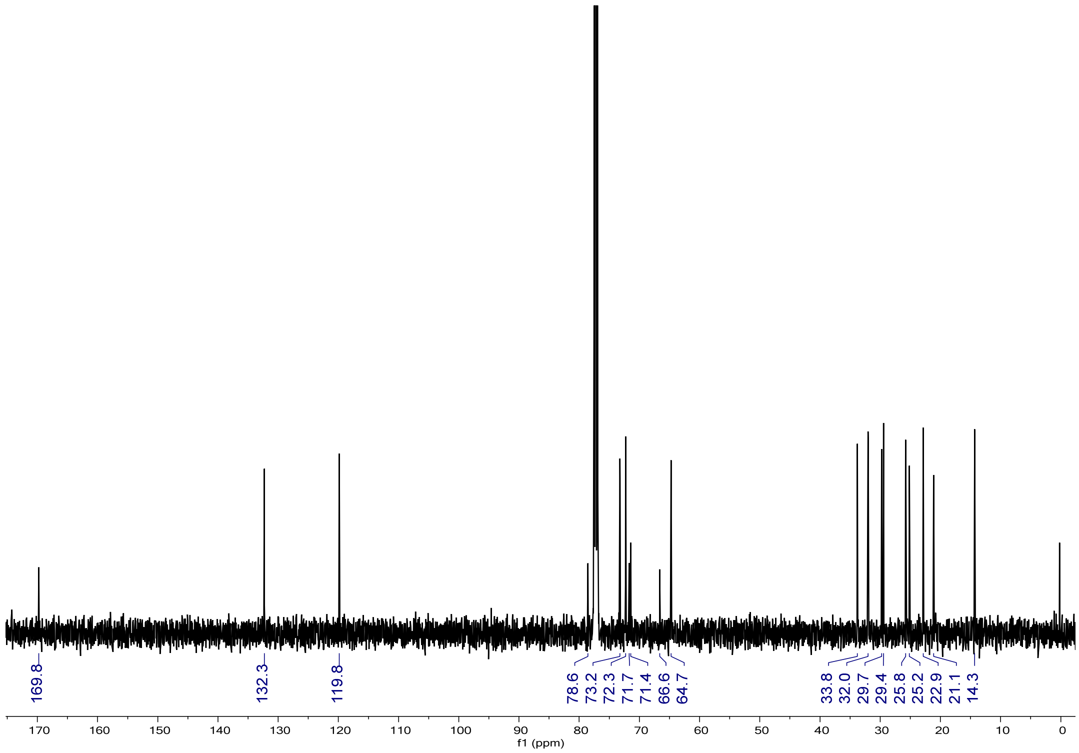

Figure S24. ^{1}H ^{13}C HSQC spectrum of compound **4**

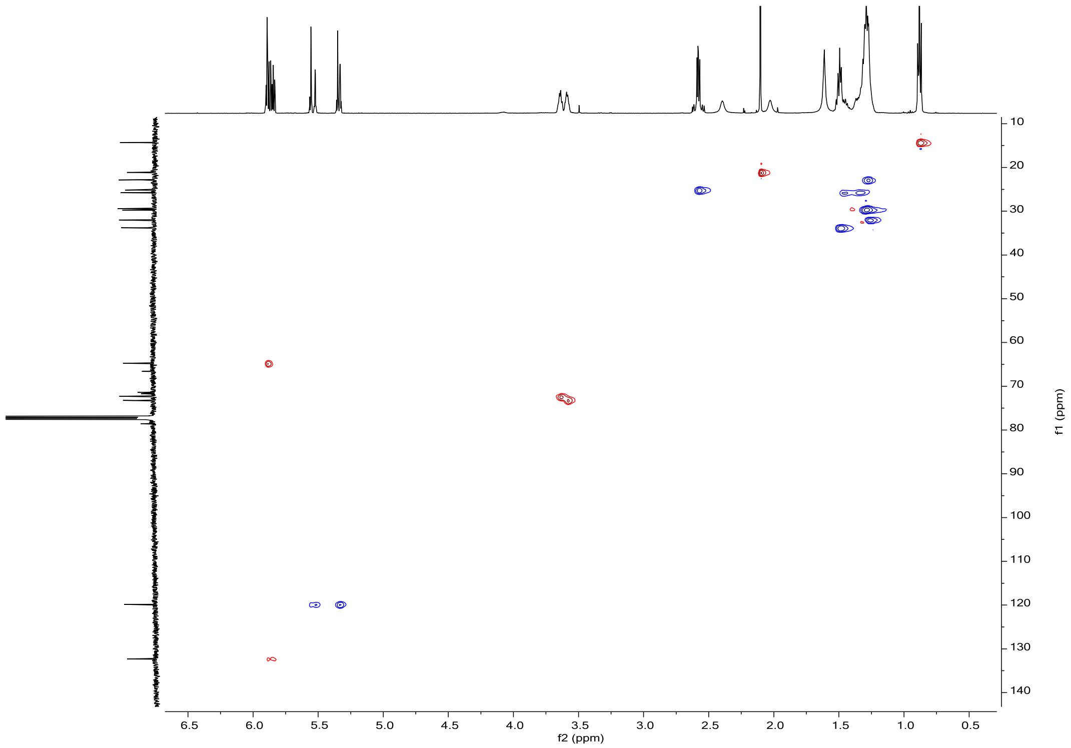

Figure S25. ^1H ^1H COSY spectrum of compound **4**

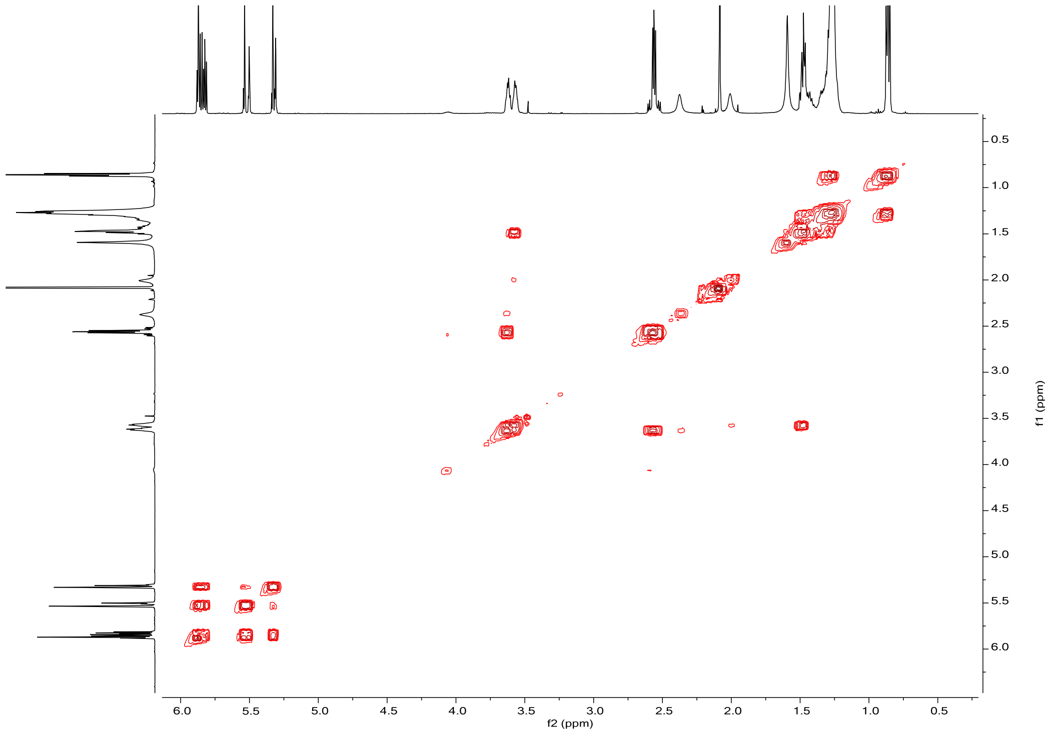

Figure S26. ^1H ^{13}C HMBC spectrum of compound **4**

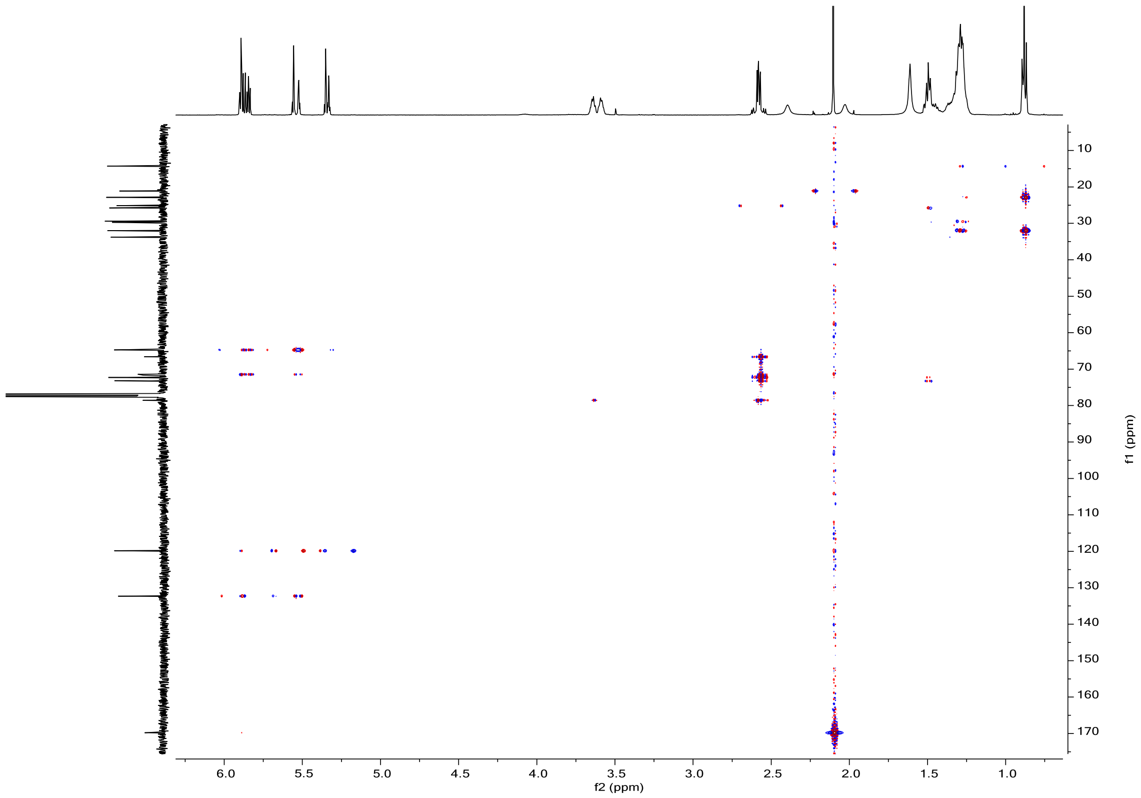

Figure S27. ^{1}H ^{1}H NOESY spectrum of compound **4**

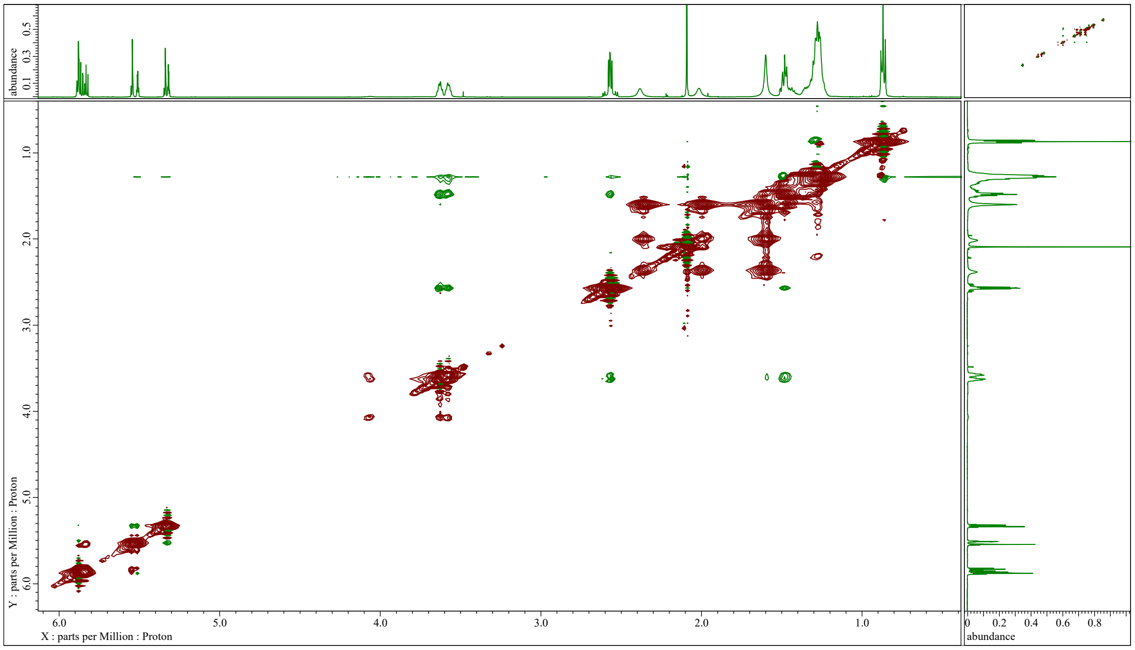

Figure S28. ^{1}H NMR spectra of compounds **11** and **4a**

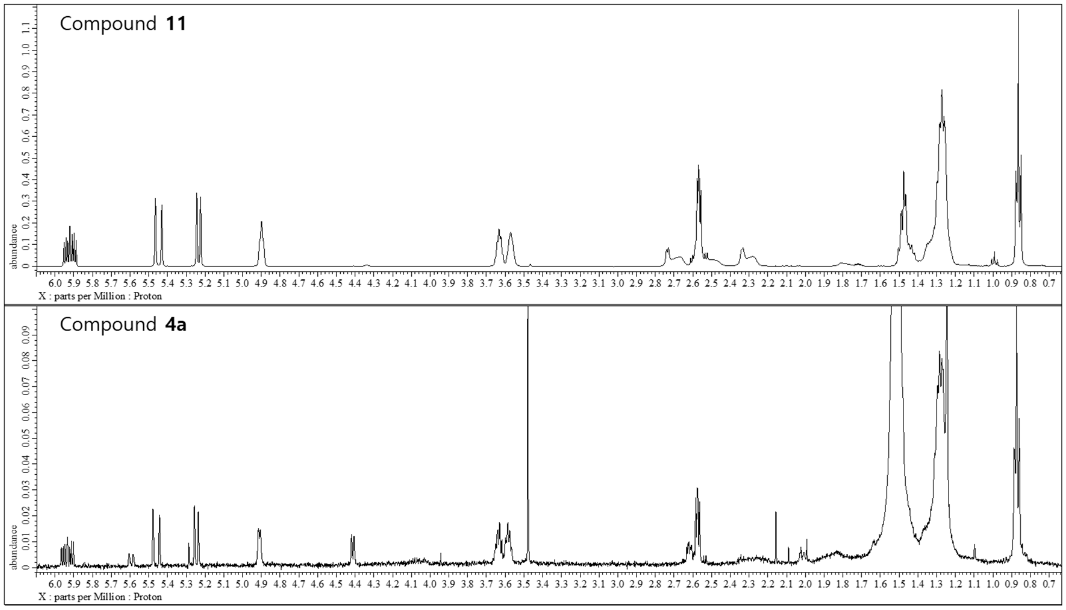

Figure S29. The effects of compounds **1−2**, **4**, **6−7**, **9−13**, and **15−16** on cell viability in epithelial ovarian cancer cells A2780

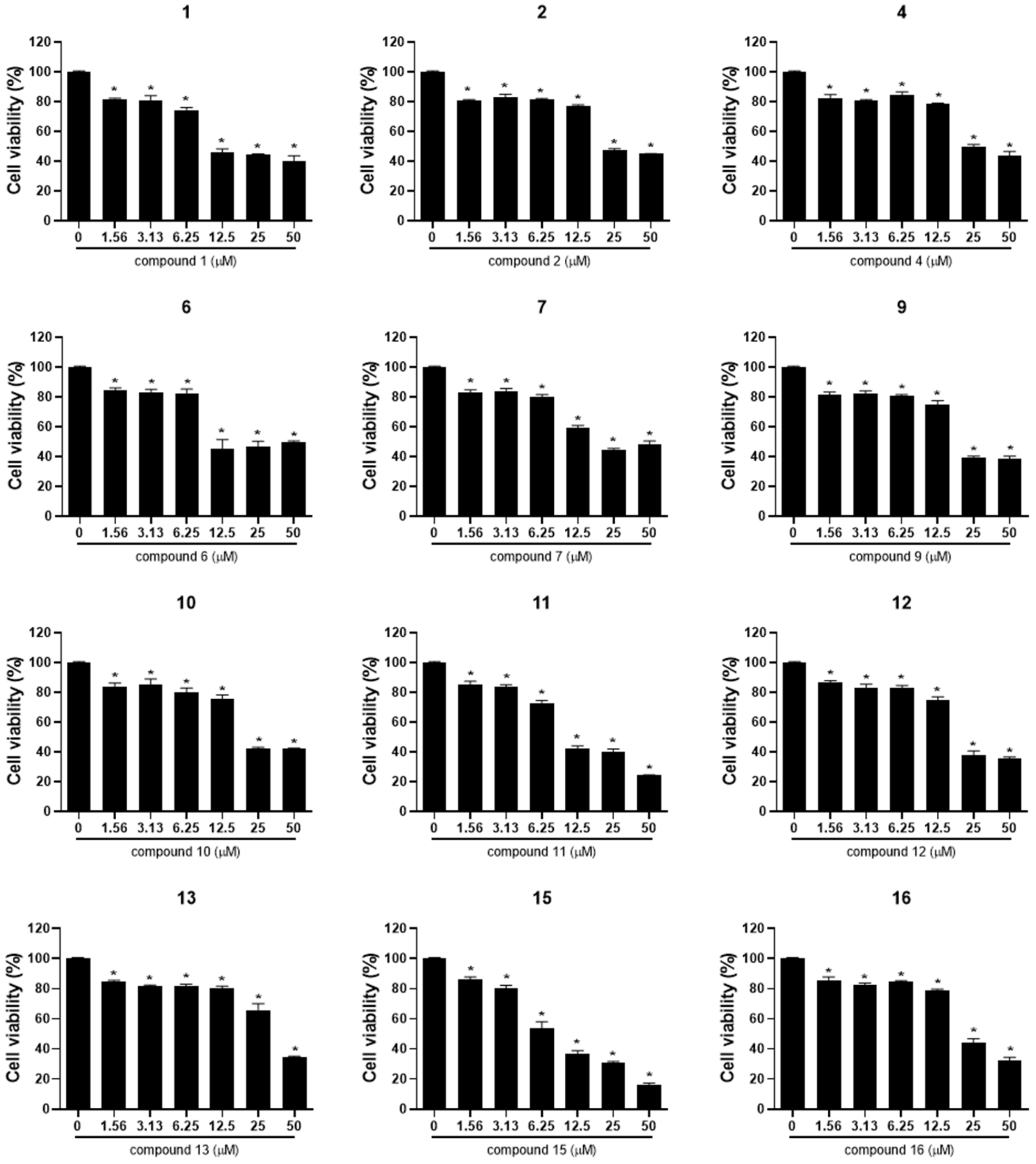

Figure S30. The effects of compounds **6**, **11**, and **15** on cell viability in epithelial ovarian cancer cells SKOV3

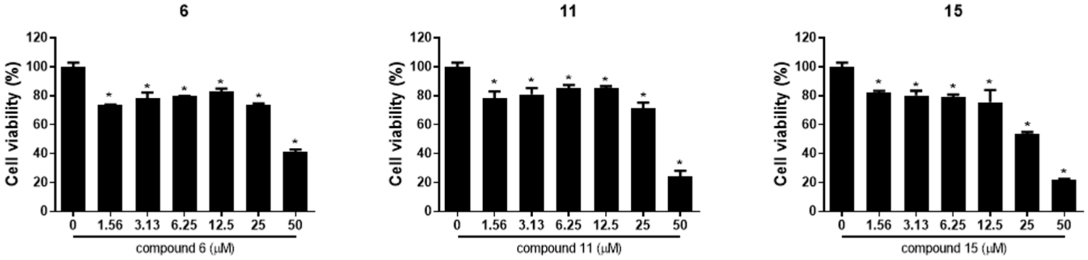

Table S1. Experimental and calculated specific rotation data of **1** and **2**

Ginsenoyne O (**1**)			Ginsenoyne P (**2**)		
Configuration	Calculated specific rotation[a]	Experimental specific rotation [b]	Configuration	Calculated specific rotation[a]	Experimental specific rotation[b]
3*S*/8*S*/9*R*/10*R*	-12.11	-21.43	3*S*/8*S*/9*R*/10*R*	-20.28	-4.90
3*R*/8*S*/9*R*/10*R*	-87.11		3*R*/8*S*/9*R*/10*R*	-98.10	
3*R*/8*R*/9*S*/10*S*	+11.42		3*R*/8*R*/9*S*/10*S*	+25.34	
3*S*/8*R*/9*S*/10*S*	+89.18		3*S*/8*R*/9*S*/10*S*	+102.61	

[a] averaged by Boltzmann weight, [b] measured at 589 nm

Table S2. The cytotoxicity of compounds **6**, 11, and **15** isolated from *P. ginseng* in RAW264.7 macrophages

Compound	IC_{50} (μM) [a]
**6**	6.28 ± 0.20
**11**	20.03 ± 0.53
**15**	18.61 ± 0.75

[a] IC_{50} value is the concentration that results in a 50% reduction in cell number when compared to control cultures.
